# Development and validation of the ISARIC 4C Deterioration model for adults hospitalised with COVID-19: a prospective cohort study

**DOI:** 10.1016/S2213-2600(20)30559-2

**Published:** 2021-04

**Authors:** Rishi K Gupta, Ewen M Harrison, Antonia Ho, Annemarie B Docherty, Stephen R Knight, Maarten van Smeden, Ibrahim Abubakar, Marc Lipman, Matteo Quartagno, Riinu Pius, Iain Buchan, Gail Carson, Thomas M Drake, Jake Dunning, Cameron J Fairfield, Carrol Gamble, Christopher A Green, Sophie Halpin, Hayley E Hardwick, Karl A Holden, Peter W Horby, Clare Jackson, Kenneth A Mclean, Laura Merson, Jonathan S Nguyen-Van-Tam, Lisa Norman, Piero L Olliaro, Mark G Pritchard, Clark D Russell, James Scott-Brown, Catherine A Shaw, Aziz Sheikh, Tom Solomon, Cathie Sudlow, Olivia V Swann, Lance Turtle, Peter J M Openshaw, J Kenneth Baillie, Malcolm G Semple, Mahdad Noursadeghi, ISARIC4C Investigators, J Kenneth Baillie, Malcolm G Semple, Peter JM Openshaw, Gail Carson, Beatrice Alex, Benjamin Bach, Wendy S Barclay, Debby Bogaert, Meera Chand, Graham S Cooke, Annemarie B Docherty, Jake Dunning, Ana da Silva Filipe, Tom Fletcher, Christopher A Green, Ewen M Harrison, Julian A Hiscox, Antonia Ying Wai Ho, Peter W Horby, Samreen Ijaz, Saye Khoo, Paul Klenerman, Andrew Law, Wei Shen Lim, Alexander J Mentzer, Laura Merson, Alison M Meynert, Mahdad Noursadeghi, Shona C Moore, Massimo Palmarini, William A Paxton, Georgios Pollakis, Nicholas Price, Andrew Rambaut, David L Robertson, Clark D Russell, Vanessa Sancho-Shimizu, Janet T Scott, Thushan de Silva, Louise Sigfrid, Tom Solomon, Shiranee Sriskandan, David Stuart, Charlotte Summers, Richard S Tedder, Emma C Thomson, AA Roger Thompson, Ryan S Thwaites, Lance CW Turtle, Maria Zambon, Hayley Hardwick, Chloe Donohue, Ruth Lyons, Fiona Griffiths, Wilna Oosthuyzen, Lisa Norman, Riinu Pius, Tom M Drake, Cameron J Fairfield, Stephen Knight, Kenneth A Mclean, Derek Murphy, Catherine A Shaw, Jo Dalton, James Lee, Daniel Plotkin, Michelle Girvan, Scott Mullaney, Claire Petersen, Egle Saviciute, Stephanie Roberts, Janet Harrison, Laura Marsh, Marie Connor, Sophie Halpin, Clare Jackson, Carrol Gamble, Gary Leeming, Andrew Law, Murray Wham, Sara Clohisey, Ross Hendry, James Scott-Brown, William Greenhalf, Victoria Shaw, Sarah McDonald, Seán Keating, Katie A. Ahmed, Jane A Armstrong, Milton Ashworth, Innocent G Asiimwe, Siddharth Bakshi, Samantha L Barlow, Laura Booth, Benjamin Brennan, Katie Bullock, Benjamin WA Catterall, Jordan J Clark, Emily A Clarke, Sarah Cole, Louise Cooper, Helen Cox, Christopher Davis, Oslem Dincarslan, Chris Dunn, Philip Dyer, Angela Elliott, Anthony Evans, Lorna Finch, Lewis WS Fisher, Terry Foster, Isabel Garcia-Dorival, Willliam Greenhalf, Philip Gunning, Catherine Hartley, Antonia Ho, Rebecca L Jensen, Christopher B Jones, Trevor R Jones, Shadia Khandaker, Katharine King, Robyn T. Kiy, Chrysa Koukorava, Annette Lake, Suzannah Lant, Diane Latawiec, L Lavelle-Langham, Daniella Lefteri, Lauren Lett, Lucia A Livoti, Maria Mancini, Sarah McDonald, Laurence McEvoy, John McLauchlan, Soeren Metelmann, Nahida S Miah, Joanna Middleton, Joyce Mitchell, Shona C Moore, Ellen G Murphy, Rebekah Penrice-Randal, Jack Pilgrim, Tessa Prince, Will Reynolds, P. Matthew Ridley, Debby Sales, Victoria E Shaw, Rebecca K Shears, Benjamin Small, Krishanthi S Subramaniam, Agnieska Szemiel, Aislynn Taggart, Jolanta Tanianis-Hughes, Jordan Thomas, Erwan Trochu, Libby van Tonder, Eve Wilcock, J. Eunice Zhang, Kayode Adeniji, Daniel Agranoff, Ken Agwuh, Dhiraj Ail, Ana Alegria, Brian Angus, Abdul Ashish, Dougal Atkinson, Shahedal Bari, Gavin Barlow, Stella Barnass, Nicholas Barrett, Christopher Bassford, David Baxter, Michael Beadsworth, Jolanta Bernatoniene, John Berridge, Nicola Best, Pieter Bothma, David Brealey, Robin Brittain-Long, Naomi Bulteel, Tom Burden, Andrew Burtenshaw, Vikki Caruth, David Chadwick, Duncan Chambler, Nigel Chee, Jenny Child, Srikanth Chukkambotla, Tom Clark, Paul Collini, Catherine Cosgrove, Jason Cupitt, Maria-Teresa Cutino-Moguel, Paul Dark, Chris Dawson, Samir Dervisevic, Phil Donnison, Sam Douthwaite, Ingrid DuRand, Ahilanadan Dushianthan, Tristan Dyer, Cariad Evans, Chi Eziefula, Chrisopher Fegan, Adam Finn, Duncan Fullerton, Sanjeev Garg, Sanjeev Garg, Atul Garg, Effrossyni Gkrania-Klotsas, Jo Godden, Arthur Goldsmith, Clive Graham, Elaine Hardy, Stuart Hartshorn, Daniel Harvey, Peter Havalda, Daniel B Hawcutt, Maria Hobrok, Luke Hodgson, Anil Hormis, Michael Jacobs, Susan Jain, Paul Jennings, Agilan Kaliappan, Vidya Kasipandian, Stephen Kegg, Michael Kelsey, Jason Kendall, Caroline Kerrison, Ian Kerslake, Oliver Koch, Gouri Koduri, George Koshy, Shondipon Laha, Steven Laird, Susan Larkin, Tamas Leiner, Patrick Lillie, James Limb, Vanessa Linnett, Jeff Little, Michael MacMahon, Emily MacNaughton, Ravish Mankregod, Huw Masson, Elijah Matovu, Katherine McCullough, Ruth McEwen, Manjula Meda, Gary Mills, Jane Minton, Mariyam Mirfenderesky, Kavya Mohandas, Quen Mok, James Moon, Elinoor Moore, Patrick Morgan, Craig Morris, Katherine Mortimore, Samuel Moses, Mbiye Mpenge, Rohinton Mulla, Michael Murphy, Megan Nagel, Thapas Nagarajan, Mark Nelson, Igor Otahal, Mark Pais, Selva Panchatsharam, Hassan Paraiso, Brij Patel, Natalie Pattison, Justin Pepperell, Mark Peters, Mandeep Phull, Stefania Pintus, Jagtur Singh Pooni, Frank Post, David Price, Rachel Prout, Nikolas Rae, Henrik Reschreiter, Tim Reynolds, Neil Richardson, Mark Roberts, Devender Roberts, Alistair Rose, Guy Rousseau, Brendan Ryan, Taranprit Saluja, Aarti Shah, Prad Shanmuga, Anil Sharma, Anna Shawcross, Jeremy Sizer, Manu Shankar-Hari, Richard Smith, Catherine Snelson, Nick Spittle, Nikki Staines, Tom Stambach, Richard Stewart, Pradeep Subudhi, Tamas Szakmany, Kate Tatham, Jo Thomas, Chris Thompson, Robert Thompson, Ascanio Tridente, Darell Tupper-Carey, Mary Twagira, Andrew Ustianowski, Nick Vallotton, Lisa Vincent-Smith, Shico Visuvanathan, Alan Vuylsteke, Sam Waddy, Rachel Wake, Andrew Walden, Ingeborg Welters, Tony Whitehouse, Paul Whittaker, Ashley Whittington, Meme Wijesinghe, Martin Williams, Lawrence Wilson, Sarah Wilson, Stephen Winchester, Martin Wiselka, Adam Wolverson, Daniel G Wooton, Andrew Workman, Bryan Yates, Peter Young

**Affiliations:** a Institute for Global Health, University College London, London, UK; b MRC Clinical Trials Unit, Institute of Clinical Trials and Methodology, University College London, London, UK; c UCL Respiratory, Division of Medicine, University College London, London, UK; d Division of Infection and Immunity, University College London, London, UK; e Centre for Medical Informatics, The Usher Institute, University of Edinburgh, Edinburgh, UK; f Department of Clinical Surgery, University of Edinburgh, Edinburgh, UK; g Queen's Medical Research Institute, University of Edinburgh, Edinburgh, UK; h School of Informatics, University of Edinburgh, Edinburgh, UK; i Department of Child Life and Health, University of Edinburgh, Edinburgh, UK; j Roslin Institute, University of Edinburgh, Edinburgh, UK; k Medical Research Council, University of Glasgow Centre for Virus Research, Glasgow, UK; l Department of Infectious Diseases, Queen Elizabeth University Hospital, Glasgow, UK; m Intensive Care Unit, Royal Infirmary Edinburgh, Edinburgh, UK; n Julius Center for Health Sciences and Primary Care, University Medical Center Utrecht, Utrecht University, Utrecht, Netherlands; o Royal Free Hospitals NHS Trust, London, UK; p Institute of Population Health Sciences, University of Liverpool, Liverpool, UK; q Liverpool Clinical Trials Centre, University of Liverpool, Liverpool, UK; r NIHR Health Protection Research Unit, Institute of Infection, Veterinary, and Ecological Sciences, Faculty of Health and Life Sciences, University of Liverpool, Liverpool, UK; s Respiratory Medicine, Alder Hey Children's Hospital, Institute in The Park, University of Liverpool, Liverpool, UK; t ISARIC Global Support Centre, Nuffield Department of Medicine, University of Oxford, Oxford, UK; u Centre for Tropical Medicine and Global Health, Nuffield Department of Medicine, University of Oxford, Oxford, UK; v National Infection Service, Public Health England, London, UK; w National Heart and Lung Institute, Imperial College London, London, UK; x Institute of Microbiology and Infection, University of Birmingham, Birmingham, UK; y Division of Epidemiology and Public Health, University of Nottingham School of Medicine, Nottingham, UK; z Walton Centre NHS Foundation Trust, Liverpool, UK; aa Health Data Research UK, London, UK; ab Tropical and Infectious Disease Unit, Royal Liverpool University Hospital, Liverpool, UK

## Abstract

**Background:**

Prognostic models to predict the risk of clinical deterioration in acute COVID-19 cases are urgently required to inform clinical management decisions.

**Methods:**

We developed and validated a multivariable logistic regression model for in-hospital clinical deterioration (defined as any requirement of ventilatory support or critical care, or death) among consecutively hospitalised adults with highly suspected or confirmed COVID-19 who were prospectively recruited to the International Severe Acute Respiratory and Emerging Infections Consortium Coronavirus Clinical Characterisation Consortium (ISARIC4C) study across 260 hospitals in England, Scotland, and Wales. Candidate predictors that were specified a priori were considered for inclusion in the model on the basis of previous prognostic scores and emerging literature describing routinely measured biomarkers associated with COVID-19 prognosis. We used internal–external cross-validation to evaluate discrimination, calibration, and clinical utility across eight National Health Service (NHS) regions in the development cohort. We further validated the final model in held-out data from an additional NHS region (London).

**Findings:**

74 944 participants (recruited between Feb 6 and Aug 26, 2020) were included, of whom 31 924 (43·2%) of 73 948 with available outcomes met the composite clinical deterioration outcome. In internal–external cross-validation in the development cohort of 66 705 participants, the selected model (comprising 11 predictors routinely measured at the point of hospital admission) showed consistent discrimination, calibration, and clinical utility across all eight NHS regions. In held-out data from London (n=8239), the model showed a similarly consistent performance (C-statistic 0·77 [95% CI 0·76 to 0·78]; calibration-in-the-large 0·00 [–0·05 to 0·05]); calibration slope 0·96 [0·91 to 1·01]), and greater net benefit than any other reproducible prognostic model.

**Interpretation:**

The 4C Deterioration model has strong potential for clinical utility and generalisability to predict clinical deterioration and inform decision making among adults hospitalised with COVID-19.

**Funding:**

National Institute for Health Research (NIHR), UK Medical Research Council, Wellcome Trust, Department for International Development, Bill & Melinda Gates Foundation, EU Platform for European Preparedness Against (Re-)emerging Epidemics, NIHR Health Protection Research Unit (HPRU) in Emerging and Zoonotic Infections at University of Liverpool, NIHR HPRU in Respiratory Infections at Imperial College London.

## Introduction

The COVID-19 pandemic has continued to overwhelm health-care systems worldwide.^1^ Effective triage of patients presenting to hospital for risk of progressive deterioration is crucial to inform clinical decision making and facilitate effective resource allocation, including hospital beds, critical care resources, and targeted drug therapies. Moreover, early identification of subgroups at higher risk of death or deterioration requiring ventilatory or critical care support enables targeted recruitment for randomised controlled trials of therapies with equipoise,^2^ and more precise delivery of treatments for which effectiveness is known to vary according to disease severity (including corticosteroids and remdesivir).^3^, ^4^, ^5^

Many multivariable clinical prognostic models for patients with COVID-19 have rapidly accrued to predict adverse outcomes of mortality or clinical deterioration.^6^ Most have been classified as being at a high risk of bias, and might not be generalisable, often because of inadequate sample sizes, reliance on single-centre data, and non-adherence to best practice methods or reporting standards during model development.^6^, ^7^ None of the multivariable prognostic models included in a systematic head-to-head external validation study outperformed univariable predictors,^8^ highlighting the need to combine large scale multisite data with rigorous model development methods to improve generalisability.

**Research in context**

**Evidence before this study**

An existing systematic review evaluated prediction models for COVID-19 indexed in PubMed, Embase, arXiv, medRxiv, and bioRxiv up to May 5, 2020. 145 models were identified, 50 of which were prognostic models seeking to predict clinical outcomes of mortality or clinical deterioration. The proposed models were considered to be poorly reported, at high risk of bias, and their reported performance was thought to be overestimated. A systematic head-to-head external validation study of 22 of these prognostic models found that none had clinical utility over and above simple univariable predictors of age for mortality and oxygen saturation for clinical deterioration. Thus, none of the multivariable models could be recommended for clinical implementation, highlighting a need for higher quality model development methodology using multicentre datasets to maximise generalisability.

**Added value of this study**

We developed and validated the 4C Deterioration model, including 11 routinely measured demographic, clinical, and laboratory predictors, for prediction of in-hospital clinical deterioration among 74 944 consecutive adults recruited to the ISARIC4C study across 260 hospitals in England, Scotland, and Wales, in accordance with TRIPOD standards. The 4C Deterioration model showed consistent discrimination, calibration, and net benefit across eight National Health Service regions during model development, with similar performance in held-out validation data from London. Importantly, the 4C Deterioration model suggested clinical utility with higher net benefit than other reproducible candidate models in a decision-curve analysis in all regions. In comparison to our recently reported 4C Mortality Score, 4C Deterioration offers significant additional value by identifying people at high risk of deterioration despite a low risk of mortality, with potential to better target interventions for those who need them and are most likely to benefit.

**Implications of all the available evidence**

Parallel prognostic models are required for the prediction of a composite outcome of clinical deterioration and of mortality alone among hospitalised adults with COVID-19. The 4C Deterioration model shows stronger potential for clinical utility and generalisability than any previous prognostic model for clinical deterioration among adults with COVID-19. The model parameters and risk prediction tool will be made freely available online alongside our previously reported 4C Mortality Score to enable independent external validation and facilitate risk stratification for therapeutic interventions.

We previously reported a pragmatic prognostic score for in-hospital mortality from the International Severe Acute Respiratory and Emerging Infections Consortium Coronavirus Clinical Characterisation Consortium (ISARIC4C) study.^9^ In this Article, we extend this work through a larger study cohort to develop and validate a prognostic model for in-hospital clinical deterioration (requirement for ventilatory support or critical care, or death). We use the wide geographical coverage of the ISARIC4C study cohort in England, Wales, and Scotland to explore between-region heterogeneity and to comprehensively assess model generalisability with respect to discrimination, calibration, and clinical utility. We have called this the 4C Deterioration model.

## Methods

### Study population and data collection

The International Severe Acute Respiratory and Emerging Infections Consortium (ISARIC)–WHO Clinical Characterisation Protocol UK (CCP-UK) study is being conducted by the ISARIC4C in 260 hospitals across England, Scotland, and Wales (National Institute for Health Research [NIHR] Clinical Research Network Central Portfolio Management System ID 14152).^10^ In this analysis, we included consecutive adults (aged ≥18 years) who had highly suspected or PCR-confirmed COVID-19. We included patients with suspected COVID-19 in the analysis because the model is intended for use in participants at the point of initial evaluation for COVID-19, when virological confirmation might not be available. The study is reported in accordance with Transparent Reporting of a multivariable prediction model for Individual Prognosis Or Diagnosis (TRIPOD) guidance.^11^ Demographic, clinical, and outcome data were collected through a publicly available standardised case record form, as reported previously.^9^, ^10^

Ethical approval was given by the South Central Oxford C research ethics committee in England (reference 13/SC/0149), and by the Scotland A research ethics committee (reference 20/SS/0028). The study is registered with ISRCTN (ISRCTN66726260).

### Outcomes

We used a composite primary outcome of in-hospital clinical deterioration, comprising any of the following: initiation of ventilatory support (non-invasive ventilation, invasive mechanical ventilation, or extracorporeal membrane oxygenation); admission to a high-dependency or intensive care unit; or death. This outcome aligns closely with a score of 6 or higher on the WHO Clinical Progression Scale^12^ and ensures that the outcome is generalisable between hospitals, since respiratory support practices can vary considerably. We included eligible participants admitted or first assessed for COVID-19 on or before Aug 26, 2020, to allow at least a 4-week interval for registration of outcome events before the final data extraction date (Sept 24, 2020). Participants who had ongoing hospital care at the end of follow-up (the point at which a final outcome was recorded in the case record form) were classified as not meeting the endpoint because the risk of deterioration declines with time since admission.^8^

### Candidate predictors

We included candidate predictors considered in our previous development and validation of the 4C Mortality Score^9^ that were available in at least 60% of the study population (appendix p 21). These predictors were specified a priori on the basis of previous prognostic scores and emerging literature describing routinely measured biomarkers associated with COVID-19 prognosis.^9^ We also included nosocomial COVID-19 acquisition to test the hypothesis that acquisition of infection in hospital might be associated with differential risk. Community-acquired infection was defined as symptom onset or first positive severe acute respiratory syndrome coronavirus 2 (SARS-CoV-2) PCR result within 7 days from admission; participants who did not meet these criteria and had either symptom onset or first positive SARS-CoV-2 PCR result more than 7 days from admission were classified as nosocomial cases.^13^ Among nosocomial cases, patients who met the deterioration outcome before the onset of COVID-19 were excluded.

Comorbidities were defined according to a modified Charlson comorbidity index,^14^ with the addition of clinician-defined obesity.^9^ We considered a composite variable representing number of comorbidities for inclusion in the model, which comprised the following comorbidities: chronic cardiac disease, chronic respiratory disease (excluding asthma), chronic renal disease, mild to severe liver disease, dementia, chronic neurological disease, connective tissue disease, diabetes, HIV or AIDS, malignancy, and clinician-defined obesity.

All predictors were taken from the day of hospital admission or the day of first clinical suspicion of COVID-19 for nosocomial cases.

### Model development

We hypothesised that heterogeneity among populations and health-care services between geographical regions might contribute to differences in model performance. Therefore, we divided the data into nine National Health Service (NHS) regions^15^ linked to contributing hospitals. Eight regions were used in model development and internal–external cross-validation (East of England, the Midlands, North East England and Yorkshire, North West England, Scotland, South East England, South West England, and Wales) as described below. The ninth region (London) was not used in model development but was held out for further validation, independent of the model training cohort.

We used a logistic regression modelling approach in view of the short time horizon for predictions (during hospital admission) and did backward elimination of the a priori candidate variables in the development cohort (appendix p 5). Continuous predictors were modelled with restricted cubic splines using a default of four knots, placed at recommended locations based on percentiles, by generating transformations using the rcs function in the rms package in R.^16^, ^17^ Glasgow coma scale scores were categorised as 15 or less than 15 because there were insufficient datapoints below 15 to fit spline functions. We used multiple imputation with chained equations to address missing data; analyses were done in each imputed dataset and pooled using Rubin's rules in the primary analysis (appendix p 5).^18^

### Model validation

During validation, we assessed model discrimination (how well predictions differentiated participants who experienced the composite outcome from those who did not, quantified as the C-statistic), calibration (agreement between predicted and observed risk, assessed using calibration slopes, calibration-in-the-large, and calibration plots) and clinical utility (quantified as net benefit).^19^ An ideal calibration slope is 1, while calibration-in-the-large should be 0 if the number of observed outcome events matches the number predicted.

The model including the selected variables was first validated in the development dataset using the internal–external cross-validation framework to concurrently examine between-region heterogeneity and assess generalisability.^19^, ^20^ In this process, each of the eight contributing NHS regions was iteratively excluded from the development set; the model was then trained using the selected predictors in the remaining regions and validated in the omitted region by quantifying the C-statistic, calibration slope, and calibration-in-the-large, and by visualisation of calibration plots (appendix p 5).^19^ We used random-effects meta-analysis to calculate pooled C-statistics, calibration slopes, and calibration-in-the-large statistics across development regions, and forest plots were examined to assess between-region heterogeneity.

The final model was then trained using the full development dataset and further validated in the held-out NHS region (London).

Decision curve analysis allows assessment of clinical utility by quantifying the trade-off between correctly identifying true positives and incorrectly identifying false positives weighted according to the threshold probability.^21^ The threshold probability represents the risk cutoff above which any given treatment or intervention might be considered, and reflects the perceived risk:benefit ratio for the intervention. Decision curve analysis was used in internal–external validation and held-out validation to quantify the net benefit of implementing the model in clinical practice,^21^ compared with the following: a treat-all approach; a treat-none approach; and using other COVID-19-specific and pre-existing prognostic models identified by recent systematic reviews.^6^, ^8^, ^9^ We included all models in which constituent variables were available in more than 60% of the cohort. Candidate models using points scores were calibrated to the validation data during decision-curve analysis, resulting in optimistic estimates of their net benefit. All decision curves were smoothed by locally weighted smoothing (LOESS) from stacked multiply imputed datasets.^19^

All analyses were done in R (version 3.6.3; appendix p 6).

### Sensitivity analysis

We assessed validation of the final model using complete case data only in the held-out NHS region. We also recalculated validation metrics when stratifying deterioration events by time to deterioration (on *vs* after day of admission or first COVID-19 assessment; and 0–3 days *vs* >3 days after admission or first COVID-19 assessment), to assess whether discrimination varied according to time interval to the outcome; when excluding participants in the validation cohort who had ongoing hospital care at the end of follow-up; when stratifying the validation cohort by community versus nosocomial infection; and when excluding community-acquired cases in which patients developed symptoms in the interval between admission and the temporal threshold for nosocomial infection, to assess any effect of incorrect inclusion of nosocomial infections within the community-acquired cases. We also repeated the analysis with an alternative multiple imputation approach, using the aregImpute function from the rms package in R,^17^ and recalculated model parameters using alternative temporal definitions of nosocomial SARS-CoV-2 infection. Finally, we assessed the discrimination of each of the continuous variables included in the final model as single univariable predictors.

### Role of the funding source

The funder of the study had no role in study design, data collection, data analysis, data interpretation, or writing of the report. All authors had full access to all the data in the study and had final responsibility for the decision to submit for publication.

## Results

Between Feb 6 and Aug 26, 2020, 74 944 eligible adults were recruited to the ISARIC4C study, of whom 66 136 (88·2%) were known to have PCR-confirmed COVID-19. Baseline demographic, physiological, and laboratory characteristics are shown stratified by outcome (table 1) and by community versus nosocomial infection (appendix p 22). Outcomes were missing for 996 (1·3%) participants. Of the 73 948 participants with outcomes available, the composite primary outcome of in-hospital clinical deterioration was met by 31 924 (43·2%), with a median time to deterioration of 4 days (IQR 1–9; appendix p 7). Risk of deterioration declined with increasing time from admission, supporting our approach to classify patients requiring ongoing hospital care at the end of follow-up as not meeting an endpoint in the primary analysis.

**Table 1:** Baseline characteristics of the study cohort, stratified by outcome

		**Overall (n=74 944)**	**Ventilatory support or HDU or ICU admission (n=15 039)**	**Death (n=16 885)**	**No deterioration (n=42 024)**	**Missing (n=996)**
Age, years		75 (60–84)	65 (55–75)	83 (77–89)	73 (57–83)	75 (59–84)
Sex						
	Female	32 807 (43·9%)	5127 (34·1%)	7106 (42·2%)	20 141 (48·0%)	433 (43·5%)
	Male	41 993 (56·1%)	9889 (65·9%)	9742 (57·8%)	21 800 (52·0%)	562 (56·5%)
	Missing	144	23	37	83	1
Ethnicity						
	White	55 016 (82·8%)	9941 (75·0%)	13 612 (89·5%)	30 854 (83·0%)	609 (79·2%)
	South Asian	3520 (5·3%)	1010 (7·6%)	479 (3·2%)	1992 (5·4%)	39 (5·1%)
	Black	2553 (3·8%)	743 (5·6%)	345 (2·3%)	1435 (3·9%)	30 (3·9%)
	East Asian	492 (0·7%)	162 (1·2%)	71 (0·5%)	255 (0·7%)	4 (0·5%)
	Other	4844 (7·3%)	1403 (10·6%)	698 (4·6%)	2656 (7·1%)	87 (11·3%)
	Missing	8519	1780	1680	4832	227
SARS-CoV-2 PCR positive		66 136 (96·9%)	13 153 (96·1%)	15 275 (98·2%)	37 106 (96·7%)	602 (97·6%)
	Missing data	6715	1346	1325	3665	379
Number of comorbidities		1 (1–2)	1 (0–2)	2 (1–3)	1 (0–2)	1 (0–2)
	Missing data	839	68	202	406	163
Nosocomial infection		7320 (9·9%)	541 (3·6%)	2093 (12·5%)	4542 (10·9%)	144 (16·6%)
	Missing data	644	64	101	351	128
Radiographic infiltrates		29 579 (61·9%)	8417 (76·9%)	6960 (63·5%)	14 015 (55·0%)	187 (53·7%)
	Missing data	27 195	4094	5920	16 533	648
Temperature, °C		37·2 (36·5–38·1)	37·5 (36·8–38·4)	37·1 (36·4–38·0)	37·1 (36·5–38·0)	37·0 (36·5–38·0)
	Missing data	3106	462	647	1783	214
Heart rate, per min		90 (78–104)	95 (82–109)	90 (77–105)	88 (76–102)	89 (79–102)
	Missing data	3383	422	717	2021	223
Respiratory rate, breaths per min		20 (18–26)	24 (20–30)	22 (18–28)	20 (18–24)	20 (18–24)
	Missing data	3535	483	687	2113	252
Systolic blood pressure, mm Hg		130 (114–147)	129 (115–145)	128 (110–147)	130 (115–147)	130 (114–148)
	Missing data	3187	426	648	1891	222
Diastolic blood pressure, mm Hg		74 (64–84)	74 (65–83)	71 (61–82)	75 (65–84)	73 (65–83)
	Missing data	3330	458	690	1955	227
Oxygen saturation, %		95 (92–97)	94 (89–96)	95 (91–97)	96 (94–97)	96 (93–98)
	Missing data	3756	537	799	2203	217
Room air or oxygen						
	Room air	48 574 (69·4%)	7213 (50·3%)	9978 (63·1%)	30 809 (78·7%)	574 (76·4%)
	Oxygen	21 453 (30·6%)	7128 (49·7%)	5824 (36·9%)	8324 (21·3%)	177 (23·6%)
	Missing data	4917	698	1083	2891	245
Glasgow coma scale		15 (15–15)	15 (15–15)	15 (15–15)	15 (15–15)	15 (15–15)
	Missing data	7839	1759	1693	4020	367
Haemoglobin, g/L		128 (112–142)	132 (116–145)	122 (105–138)	129 (113–142)	128 (112–141)
	Missing data	11 748	1398	2562	7448	340
White cell count, × 10^9^ cells per L		7·5 (5·4–10·7)	8·0 (5·7–11·2)	8·3 (5·8–11·9)	7·1 (5·2–9·9)	7·7 (5·4–10·7)
	Missing data	12 130	1491	2669	7623	347
Lymphocytes, × 10^9^ cells per L		0·90 (0·60–1·30)	0·80 (0·60–1·20)	0·80 (0·50–1·16)	0·96 (0·67–1·40)	0·90 (0·60–1·30)
	Missing data	12345	1535	2700	7763	347
Neutrophils, × 10^9^ cells per L		5·8 (3·9–8·7)	6·4 (4·3–9·3)	6·6 (4·3–9·9)	5·3 (3·6–7·9)	5·8 (3·7–8·7)
	Missing data	12 308	1533	2702	7726	347
Platelets, × 10^9^ cells per L		221 (167–290)	219 (166–287)	209 (154–284)	226 (173–294)	218 (161–285)
	Missing data	12 463	1538	2730	7847	348
Alanine aminotransferase, IU/L		25 (16–43)	33 (21–54)	22 (15–37)	24 (15–40)	24 (15–43)
	Missing data	26 735	3861	6289	16 085	500
Bilirubin, mg/dL		10 (7–14)	10 (7–15)	10 (7–15)	9 (6–13)	10 (7–14)
	Missing data	22 931	3107	5308	14 025	491
Urea, mmol/L		7 (5–11)	7 (5–11)	10 (7–16)	6 (4–9)	7 (5–12)
	Missing data	18 509	2761	3965	11 344	439
Creatinine, μmol/L		86 (67–121)	86 (68–118)	106 (76–158)	81 (64–107)	85 (68–122)
	Missing data	12 656	1609	2699	7998	350
Sodium, mmol/L		137 (134–140)	136 (133–139)	138 (135–143)	137 (134–140)	137 (134–140)
	Missing data	12 277	1491	2623	7810	353
C-reactive protein, mg/L		80 (33–154)	126 (64–210)	98 (48–174)	58 (22–119)	76 (30–151)
	Missing data	16 318	2198	3508	10 202	410
NHS region						
	East of England	7852 (10·5%)	1640 (10·9%)	1935 (11·5%)	4223 (10·0%)	54 (5·4%)
	London	8239 (11·0%)	2275 (15·1%)	1509 (8·9%)	4400 (10·5%)	55 (5·5%)
	Midlands	15 583 (20·8%)	2547 (16·9%)	3699 (21·9%)	9068 (21·6%)	269 (27·0%)
	North East and Yorkshire	10 305 (13·8%)	2233 (14·8%)	2223 (13·2%)	5773 (13·7%)	76 (7·6%)
	North West	12 914 (17·2%)	2170 (14·4%)	3290 (19·5%)	7311 (17·4%)	143 (14·4%)
	Scotland	3066 (4·1%)	605 (4·0%)	572 (3·4%)	1846 (4·4%)	43 (4·3%)
	South East	9445 (12·6%)	2130 (14·2%)	1971 (11·7%)	5051 (12·0%)	293 (29·4%)
	South West	3915 (5·2%)	723 (4·8%)	794 (4·7%)	2361 (5·6%)	37 (3·7%)
	Wales	3625 (4·8%)	716 (4·8%)	892 (5·3%)	1991 (4·7%)	26 (2·6%)

Data are median (IQR) or n (%), calculated from non-missing data. Participants are shown by the first chronological deterioration category through which they met the composite primary outcome (HDU or ICU admission, ventilatory support, or death). HDU=high-dependency unit. ICU=intensive care unit. SARS-CoV-2=severe acute respiratory syndrome coronavirus 2. NHS=National Health Service.

66 705 participants from eight regions (with sample sizes ranging from 3066 to 15 583) were included in model development, with a further 8239 from one region (London) held out for additional validation. Candidate predictors and their proportions of missingness, stratified by NHS region, are shown in the appendix (p 8)). Proportions of missingness were similar across regions for each variable.

**Figure 1:**
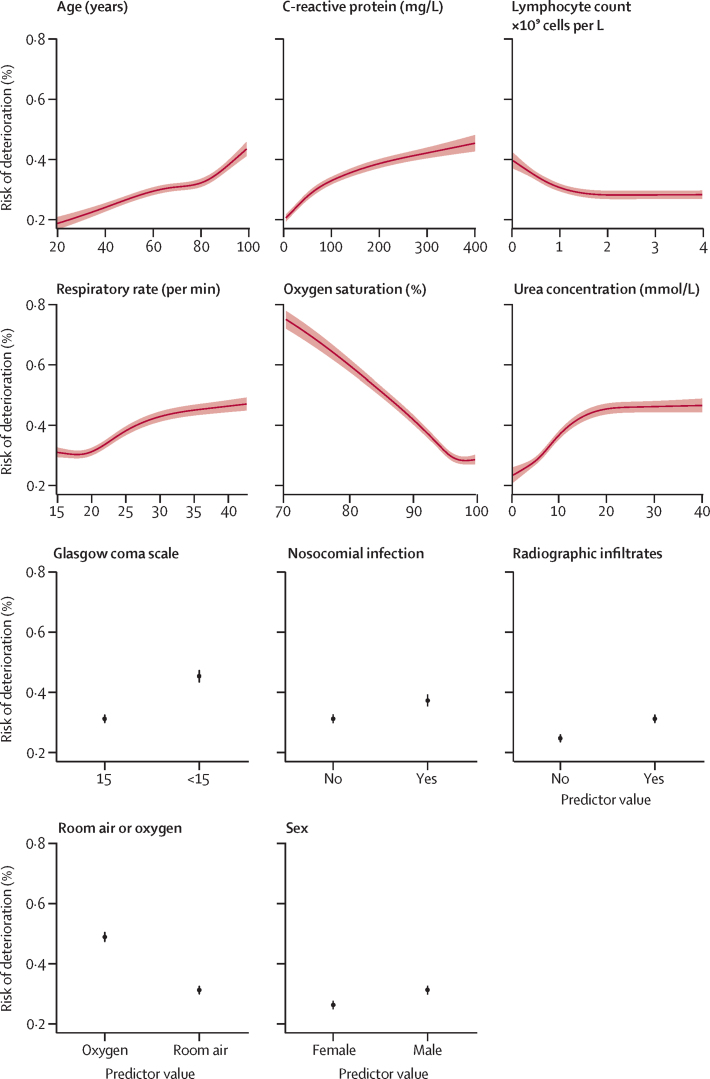
Multivariable associations between selected predictors and outcome in final model Variable selection was done in each imputed dataset with backward elimination within each National Health Service region (appendix p 5). Black lines and dots indicate point estimates; red shaded regions and error bars indicate 95% CIs.

In backward elimination, 11 predictors were retained in more than five (>50%) of ten multiply imputed datasets in more than four (>50%) of eight development NHS regions, and entered the final model. These predictors were age, sex, nosocomial infection, Glasgow coma scale score, peripheral oxygen saturation (SpO_2_) at admission, breathing room air or oxygen therapy (contemporaneous with SpO_2_ measurement), respiratory rate, urea concentration, C-reactive protein concentration, lymphocyte count, and presence of radiographic chest infiltrates. Associations (including non-linearities) between each predictor and the outcome from the final 4C Deterioration model trained on the full development cohort are shown in figure 1, and full model coefficients are presented in the appendix (p 23)) to enable independent model reconstruction.

**Figure 2:**
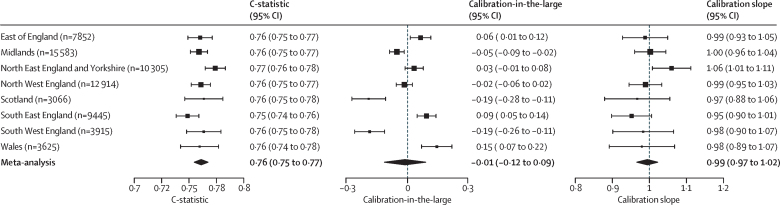
Internal–external cross-validation of selected model by National Health Service region Dashed lines indicate lines of perfect calibration-in-the-large (0) and calibration slope (1). Black squares indicate point estimates; bars indicate 95% CIs; diamonds indicate pooled estimates from a random-effects meta-analysis (n=66 705).

Forest plots showing model discrimination (C-statistic) and calibration metrics (slope and calibration-in-the-large) from internal–external cross-validation^20^ of the prognostic model in the development cohort are shown in figure 2. C-statistics were consistent across development NHS regions (point estimates 0·75 to 0·77; pooled random-effects meta-analysis estimate 0·76 [95% CI 0·75 to 0·77]). Calibration slopes were also consistent across regions, with little evidence of heterogeneity (point estimates 0·95 to 1·06; pooled estimate 0·99 [0·97 to 1·02]). There was minor heterogeneity across NHS regions in calibration-in-the-large, probably reflecting some variation in baseline risk between regions (point estimates –0·19 to 0·15; pooled estimate –0·01 [–0·12 to 0·09]). Overall risk was slightly underestimated in South East England (calibration-in-the-large 0·09 [95% CI 0·05 to 0·14]) and Wales (0·15 [0·07 to 0·22]), and overestimated in Scotland (−0·19 [–0·28 to –0·11]) and South West England (−0·19 [–0·26 to –0·11]). Pooled calibration plots by NHS region are shown in the appendix (p 9)). In view of the minor variation in calibration-in-the-large between NHS regions, we also repeated internal–external cross-validation with recalibration to each NHS region by re-estimation of the model intercept; calibration plots with recalibrated intercepts confirmed small improvements in model calibration (appendix p 10).

Decision-curve analyses in the validation sets from internal–external cross-validation, without recalibration of the new model, are shown in the appendix (p 11)), with benchmarking to 11 existing candidate prognostic models for which the constituent variables were available in more than 60% of participants. The 4C Deterioration model had higher net benefit than any of the existing models and the treat-all or treat-none strategies across a broad range of threshold probabilities in all development NHS regions (without local recalibration).

**Figure 3:**
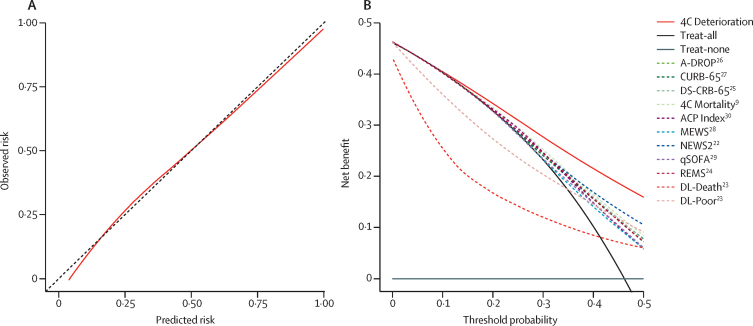
Calibration and decision-curve analysis in held-out London region (n=8239) (A) Calibration is shown using locally weighted smoothing (LOESS) across multiply imputed datasets. (B) Net benefit is shown with LOESS for each candidate model compared with the treat-all and treat-none approaches. Points score models are recalibrated to the validation data, resulting in optimistic estimates of net benefit for these models. NEWS2=National Early Warning Score 2. REMS=Rapid Emergency Medicine Score. MEWS=Modified Early Warning Score. qSOFA=quick Sequential Organ Failure Assessment. ACP=Age and C-reactive Protein.

Subsequently, we validated the final prognostic model, trained on the full development cohort, in the held-out NHS region. Discrimination and calibration metrics for the 4C Deterioration model were similar to the estimates from internal–external cross-validation (table 2), with C-statistic 0·77 (0·76 to 0·78), calibration-in-the-large 0·00 (−0·05 to 0·05), and calibration slope 0·96 (0·91 to 1·01; visual calibration curve shown in figure 3A). Discrimination was higher for the 4C Deterioration model than for the other existing candidates. The sensitivity, specificity, positive-predictive value, and negative-predictive value across the full range of probability thresholds from the model are shown in the appendix (p 12)).

**Table 2:** Validation performance in held-out London region

	Original outcome*	**C-statistic**	**Calibration-in-the-large**	**Slope**
4C Deterioration	Deterioration (in-hospital)	0·77 (0·76 to 0·78)	0·00 (−0·05 to 0·05)	0·96 (0·91 to 1·01)
NEWS2^22^	Deterioration (1 day)	0·69 (0·68 to 0·70)	..	..
4C Mortality^9^	Mortality (in-hospital)	0·68 (0·67 to 0·69)	..	..
DL-Death^23^	Mortality (in-hospital)	0·67 (0·66 to 0·69)	2·24 (2·18 to 2·30)	0·14 (0·10 to 0·18)
DL-Poor^23^	Deterioration (in-hospital)	0·67 (0·66 to 0·68)	0·53 (0·47 to 0·59)	0·13 (0·10 to 0·16)
REMS^24^	Mortality (in-hospital)	0·66 (0·65 to 0·68)	..	..
DS CRB-65^25^	Mortality (30 days)	0·66 (0·65 to 0·67)	..	..
A-DROP^26^	Mortality (30 days)	0·65 (0·64 to 0·66)	..	..
CURB-65^27^	Mortality (30 days)	0·65 (0·64 to 0·66)	..	..
MEWS^28^	Deterioration (in-hospital)	0·63 (0·62 to 0·64)	..	..
qSOFA^29^	Mortality (in-hospital)	0·63 (0·62 to 0·64)	..	..
ACP Index^30^	Mortality (12 days)	0·62 (0·61 to 0·63)	..	..

Models are shown for prediction of in-hospital clinical deterioration and are sorted by C-statistic (total sample size 8239 participants). Calibration-in-the-large and slopes are not shown for points score models because they are not on a probability scale. NEWS2=National Early Warning Score 2. REMS=Rapid Emergency Medicine Score. MEWS=Modified Early Warning Score. qSOFA=quick Sequential Organ Failure Assessment. ACP=Age and C-reactive Protein.

* Original intended outcome for each candidate model during development.

Decision-curve analysis in the held-out NHS region to further examine clinical utility for the 4C Deterioration model showed higher net benefit than all other candidates and the treat-all and treat-none approaches across a range of threshold probabilities (figure 3B).

**Figure 4:**
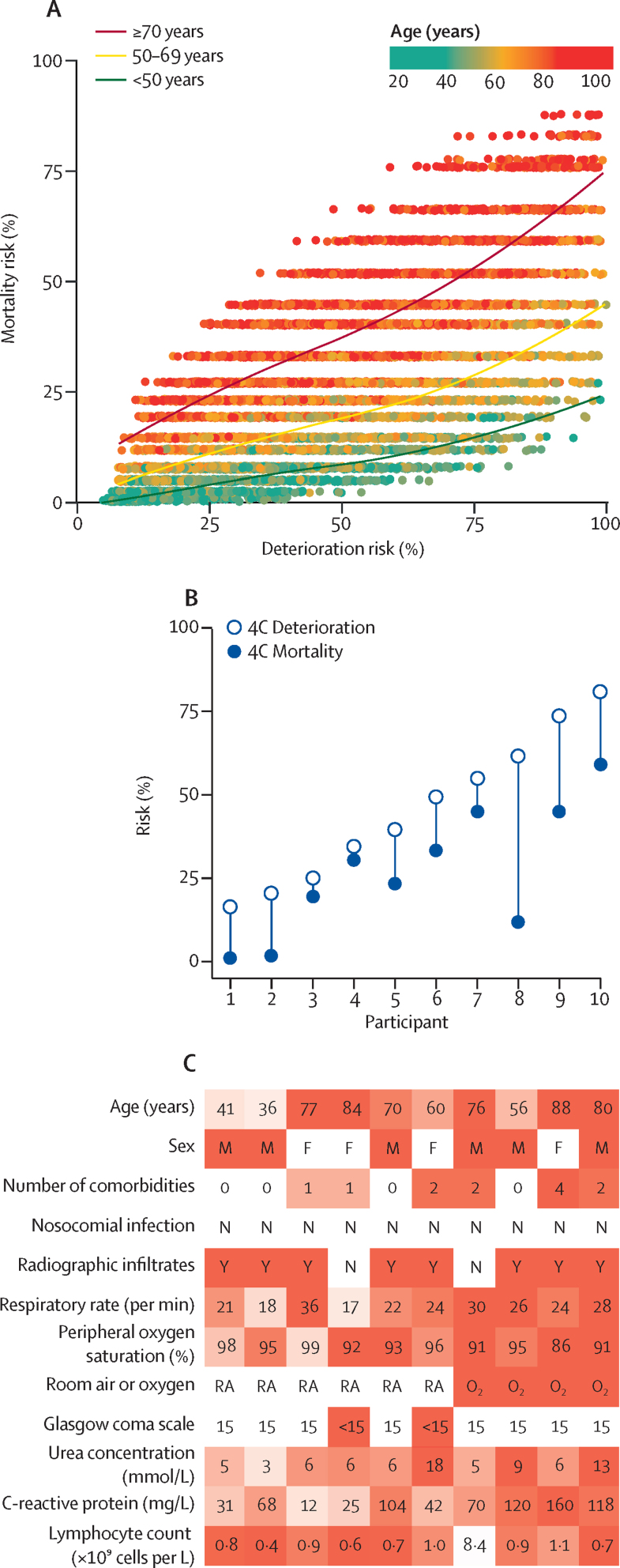
4C Deterioration versus 4C Mortality predictions for London validation cohort (n=8239) and randomly sampled example patients (n=10) 4C Mortality probabilities are calculated from points scores, based on observed mortality risk for each score in the original validation data. (A) Smoothed plot reflects locally estimated scatterplot smoothing fit, stratified by age (<50 years, 50–69 years, or ≥70 years), in the London cohort (n=8239). (B) Ten example patients were randomly sampled from the validation cohort, stratified by deciles of 4C Deterioration model predictions. (C) Characteristics of each example participant, with red indicating characteristics associated with higher risk predictions. M=male. F=female. N=no. Y=yes. RA=breathing room air. O_2_=receiving oxygen therapy.

We anticipate that clinicians might wish to evaluate risk of deterioration or death separately. Therefore, for illustration, we compared predictions from the 4C Deterioration model to our previously reported 4C Mortality Score^9^ in the London validation cohort, stratified by age (figure 4A), sex, and ethnicity (appendix p 13). In addition, ten example participants selected at random from each decile of 4C Deterioration predictions in the London cohort are shown in figure 4B, with their clinical characteristics summarised in figure 4C. Overall, deterioration predictions appeared appropriately higher than those for mortality. Importantly, the covariance between 4C Mortality Score and 4C Deterioration predictions was lower among younger age groups, among whom discrepancies between predictions were therefore greater. There were no differences in covariance by sex or ethnicity after stratification by age.

Validation of the model in complete case data from the held-out London region showed similar results to the primary analyses (appendix p 24). Stratification of outcome events (on *vs* after day of admission or first COVID-19 assessment; and 0–3 days *vs* >3 days after admission or first COVID-19 assessment) in the London validation cohort resulted in slightly lower C-statistics with longer time horizons for the 4C Deterioration model and most other models (appendix p 26). However, for some models in which mortality was the original intended outcome (including the 4C Mortality model), discrimination appeared better over the longer time horizons. Validation metrics in the London cohort were similar to those of the primary analysis when excluding participants who had ongoing hospital care at the end of follow-up (appendix p 27), when restricted to community-acquired infections (appendix p 28), and when community-acquired infections with symptom onset after admission were excluded (appendix p 29). Among nosocomial cases, the C-statistic was slightly lower for the 4C Deterioration model than for the primary analysis (0·73 [0·68 to 0·78]), although discrimination remained higher than that of the other candidate models, and calibration-in-the-large was 0·32 (0·12 to 0·53), suggesting elevated baseline risk among participants with nosocomial infection (appendix p 28). Repeating the analyses with use of an alternative multiple-imputation approach and with shorter and longer temporal definitions of nosocomial infection led to similar results to the primary analysis (appendix pp 14–19, 30). Of the continuous variables in the final model, serum C-reactive protein concentration was the strongest univariable predictor for deterioration (C-statistic 0·68 [0·66 to 0·69]) but had lower discrimination than the full multivariable model (appendix p 31).

## Discussion

We developed and validated a prognostic model for in-hospital clinical deterioration among 74 944 consecutive adults hospitalised with COVID-19 and recruited to the ISARIC4C study across 260 hospitals in England, Scotland, and Wales. The final model integrates 11 routinely available predictors and is intended for use at the point of admission for community-acquired cases, or first evaluation of suspected nosocomial COVID-19. Internal–external cross-validation showed consistent discrimination, calibration, and net benefit across NHS regions, which were confirmed in further validation in the held-out London region. The model provides a probability output that indicates the chance of the individual under evaluation having the outcome. These predictions will enable clinicians to objectively assess deterioration risk to inform the need for interventions such as ongoing hospital admission, consideration for critical care, and initiation of therapeutic agents. Importantly, the 4C Deterioration model achieved higher net benefit than other candidate risk-stratification tools across a broad range of risk thresholds in all NHS regions. Thus, the 4C Deterioration model has strong potential for clinical utility and generalisability.

Our 4C Deterioration model can be implemented programmatically alongside our previously reported 4C Mortality Score.^9^ Covariance between the 4C Deterioration and 4C Mortality predictions was not systematically different by sex or ethnicity, but was attenuated among younger age groups. Thus, the greatest discordance of risks is evident in younger patients. This finding suggests that younger people who deteriorated were more likely to have escalation of treatment through admission to a high-dependency or intensive care unit or through ventilatory support, whereas older people who deteriorated were more likely to die. These observations might be mediated, in part, by differential treatment escalation decisions associated with age. Moreover, our comparison of the models for ten randomly selected patients across the distribution of outcome risks illustrates examples of cases with relatively low risks of death, but moderate to high risks of deterioration. These discordances underline the need for parallel prognostic models for a composite outcome of clinical deterioration and for mortality alone. Notably, the discrimination of the 4C Deterioration declined slightly with increasing time to outcome events, whereas that of the 4C Mortality model improved, probably reflecting the fact that most deterioration events that occurred more than 3 days after admission were deaths, whereas earlier events were more likely to be initiations of ventilatory support or high-dependency or intensive care. Application of the 4C Mortality Score and 4C Deterioration model together therefore provides the optimal approach for clinicians to predict the appropriate outcome as required to inform clinical management decisions.

We overcame the weaknesses of previous COVID-19 predictive models^6^, ^8^ by adhering to TRIPOD standards^11^ and retaining continuous variables without arbitrary categorisation, while accounting for non-linear associations, to avoid loss of information.^31^ Moreover, we used the largest dataset to date, to our knowledge, to develop and validate the 4C Deterioration model, reducing the risk of overfitting due to inadequate sample size. We exploited the wide geographical coverage across nine NHS regions in England, Scotland, and Wales to explore between-region heterogeneity in model performance using internal–external cross-validation.^32^ Although discrimination, calibration slopes, and net benefit were largely very consistent, we noted minor variation in calibration-in-the-large, suggesting some variation in baseline risk between regions. Our approach of recalibrating the model intercept to each NHS region showed the potential to address such heterogeneity and could be used to update the model if risk changes temporally (as novel therapies are implemented) and among different populations. Nonetheless, net benefit, which accounts for model discrimination and calibration in quantifying clinical utility, was higher for the 4C Deterioration model than for all other candidates, even without recalibration, across all NHS regions and in the held-out validation dataset. This was the case even when comparing to points-based models, which might have achieved overly optimistic performance in decision-curve analyses because they were recalibrated to the validation datasets. We also used a best-practice approach to missing data with multiple imputation,^33^ and obtained consistent results with an alternative imputation approach.

Our 4C Deterioration model was developed and validated in the context of current care; predictions should therefore be interpreted as reflecting both baseline risk and potential mitigation through in-hospital interventions. Ongoing prospective external validation of the 4C Deterioration model will be required to consider the need for temporal recalibration^34^ and to evaluate model performance in diverse international settings outside of the ISARIC4C study. Although the model showed consistent performance across England, Wales, and Scotland, validation in other counties should be prioritised to enable its clinical implementation internationally. We have provided the underlying model coefficients to enable this. Another limitation is that we only included predictors that were routinely measured as part of clinical care during the study period, and specified that they had to be available among more than 60% of the population for inclusion in the analysis. Thus, we were unable to assess candidate models that include predictors such as lactate dehydrogenase or D-dimer concentrations, because these variables were only available in a small proportion of participants. Future studies could consider standardised capture of laboratory measurements considered to have prognostic value to enable inclusion of these variables in model development and validation at scale. Moreover, we note that novel molecular biomarkers currently under investigation might also offer prognostic value.^35^ Blood transcript, protein, and metabolite measurements will be available from a subset of the ISARIC4C participants and could be integrated into risk-stratification tools in future studies.

In summary, we present a prognostic model for clinical deterioration among hospitalised adults with community-acquired or hospital-acquired COVID-19, validated in nine NHS regions in England, Scotland, and Wales. The model uses readily available clinical predictors to predict the probability of in-hospital deterioration and will be made freely available online alongside our previously reported mortality risk score,^9^ to inform clinical decision making and patient stratification for therapeutic interventions.

## Data sharing

Access to all data and samples collected by ISARIC4C are controlled by an Independent Data and Materials Access Committee composed of representatives of research funders, academia, clinical medicine, public health, and industry. The application process for access to the data is available on the ISARIC4C website.

## References

[1] WHO (Sept 14, 2020). Coronavirus disease 2019 (COVID-19) weekly epidemiological update. https://www.who.int/emergencies/diseases/novel-coronavirus-2019/situation-reports.

[2] Furlow B (2020). COVACTA trial raises questions about tocilizumab's benefit in COVID-19. Lancet Rheumatol.

[3] Beigel JH, Tomashek KM, Dodd LE (2020). Remdesivir for the treatment of Covid-19—final report. N Engl J Med.

[4] Sterne JAC, Murthy S, Diaz J V (2020). Association between administration of systemic corticosteroids and mortality among critically ill patients with COVID-19. JAMA.

[5] The RECOVERY Collaborative Group (2020). Dexamethasone in hospitalized patients with COVID-19—preliminary report. N Engl J Med.

[6] Wynants L, Van Calster B, Collins GS (2020). Prediction models for diagnosis and prognosis of COVID-19 infection: systematic review and critical appraisal. BMJ.

[7] Wolff RF, Moons KGM, Riley RD (2019). PROBAST: a tool to assess the risk of bias and applicability of prediction model studies. Ann Intern Med.

[8] Gupta RK, Marks M, Samuels THA (2020). Systematic evaluation and external validation of 22 prognostic models among hospitalised adults with COVID-19: an observational cohort study. Eur Respir J.

[9] Knight SR, Ho A, Pius R (2020). Risk stratification of patients admitted to hospital with covid-19 using the ISARIC WHO Clinical Characterisation Protocol: development and validation of the 4C Mortality Score. BMJ.

[10] Docherty AB, Harrison EM, Green CA (2020). Features of 20 133 UK patients in hospital with covid-19 using the ISARIC WHO Clinical Characterisation Protocol: prospective observational cohort study. BMJ.

[11] Collins GS, Reitsma JB, Altman DG, Moons KGM (2015). Transparent reporting of a multivariable prediction model for individual prognosis or diagnosis (TRIPOD): the TRIPOD statement. BMJ.

[12] WHO Working Group on the Clinical Characterisation and Management of COVID-19 infection (2020). A minimal common outcome measure set for COVID-19 clinical research. Lancet Infect Dis.

[13] European Centre for Disease Prevention and Control Surveillance definitions for COVID-19. https://www.ecdc.europa.eu/en/covid-19/surveillance/surveillance-definitions.

[14] Charlson ME, Pompei P, Ales KL, MacKenzie CR (1987). A new method of classifying prognostic comorbidity in longitudinal studies: development and validation. J Chronic Dis.

[15] NHS Regional teams. https://www.england.nhs.uk/about/regional-area-teams/.

[16] Frank E (2004). Harrell. Biostatistical Modeling. https://hbiostat.org/doc/bbr.pdf.

[17] Harrell FE (2019). rms: Regression modeling strategies. https://cran.r-project.org/web/packages/rms/index.html.

[18] van Buuren S, Groothuis-Oudshoorn K (2011). mice: Multivariate Imputation by Chained Equations in R. J Stat Softw.

[19] Steyerberg EW, Vergouwe Y (2014). Towards better clinical prediction models: seven steps for development and an ABCD for validation. Eur Heart J.

[20] Debray TPA, Riley RD, Rovers MM, Reitsma JB, Moons KGM, Cochrane IPD (2015). Individual participant data (IPD) meta-analyses of diagnostic and prognostic modeling studies: guidance on their use. PLoS Med.

[21] Vickers AJ, van Calster B, Steyerberg EW (2019). A simple, step-by-step guide to interpreting decision curve analysis. Diagn Progn Res.

[22] Smith GB, Prytherch DR, Meredith P, Schmidt PE, Featherstone PI (2013). The ability of the National Early Warning Score (NEWS) to discriminate patients at risk of early cardiac arrest, unanticipated intensive care unit admission, and death. Resuscitation.

[23] Zhang H, Shi T, Wu X (2020). Risk prediction for poor outcome and death in hospital in-patients with COVID-19: derivation in Wuhan, China and external validation in London, UK. medRxiv.

[24] Olsson T, Terent A, Lind L (2004). Rapid Emergency Medicine score: a new prognostic tool for in-hospital mortality in nonsurgical emergency department patients. J Intern Med.

[25] Dwyer R, Hedlund J, Henriques-Normark B, Kalin M (2014). Improvement of CRB-65 as a prognostic tool in adult patients with community-acquired pneumonia. BMJ Open Respir Res.

[26] Miyashita N, Matsushima T, Oka M (2006). The JRS guidelines for the management of community-acquired pneumonia in adults: an update and new recommendations. Intern Med.

[27] Lim WS, van der Eerden MM, Laing R (2003). Defining community acquired pneumonia severity on presentation to hospital: an international derivation and validation study. Thorax.

[28] Subbe CP, Kruger M, Rutherford P, Gemmel L (2001). Validation of a modified Early Warning Score in medical admissions. QJM.

[29] Seymour CW, Liu VX, Iwashyna TJ (2016). Assessment of clinical criteria for sepsis: for the third international consensus definitions for sepsis and septic shock (Sepsis-3). JAMA.

[30] Lu J, Hu S, Fan R (2020). ACP risk grade: a simple mortality index for patients with confirmed or suspected severe acute respiratory syndrome coronavirus 2 disease (COVID-19) during the early stage of outbreak in Wuhan, China. medRxiv.

[31] Royston P, Altman DG, Sauerbrei W (2006). Dichotomizing continuous predictors in multiple regression: a bad idea. Stat Med.

[32] Steyerberg EW, Harrell FE (2016). Prediction models need appropriate internal, internal-external, and external validation. J Clin Epidemiol.

[33] Riley RD, van der Windt D, Croft P, Moons KGM (2019). Prognosis research in healthcare: concepts, methods, and impact.

[34] Booth S, Riley RD, Ensor J, Lambert PC, Rutherford MJ (2020). Temporal recalibration for improving prognostic model development and risk predictions in settings where survival is improving over time. Int J Epidemiol.

[35] Del Valle DM, Kim-Schulze S, Huang HH (2020). An inflammatory cytokine signature predicts COVID-19 severity and survival. Nat Med.

