# Supplementary material for: Development and validation of the ISARIC 4C Deterioration model for adults hospitalised with COVID-19: a prospective cohort study
Source: Lancet Respir Med. 2021 Apr;9(4):349–59. doi: 10.1016/S2213-2600(20)30559-2 (PMC7832571; doi:10.1016/S2213-2600(20)30559-2)
Supplement: Supplementary appendix [file mmc1.pdf]

THE LANCET
Respiratory Medicine

Supplementary appendix

This appendix formed part of the original submission and has been peer reviewed. We post it as supplied by the authors.

Supplement to: Gupta RK, Harrison EM, Ho A, et al. Development and validation of the ISARIC 4C Deterioration model for adults hospitalised with COVID-19: a prospective cohort study. *Lancet Respir Med* 2021; published online Jan 11. http://dx.doi.org/10.1016/S2213-2600(20)30559-2.

Development and validation of the 4C Deterioration model for adults hospitalised with COVID-19: a prospective cohort study using the ISARIC WHO Clinical Characterisation Protocol.

Supplementary Appendix

Contents

ISARIC4C Investigators 3

Supplementary Methods 5

Variable selection 5

Missing data 5

Validation metrics........ 5

Software 6

Sample size........ 6

Supplementary Results 7

Supplementary Figure 1: Days to deterioration following admission, stratified by first chronological type of deterioration........ 7

Supplementary Figure 2: Heatmap showing missingness (%) of candidate predictors and outcome, stratified by NHS region (n = 74,944 participants)........ 8

Supplementary Figure 3: Pooled calibration across multiple imputed datasets during IECV (a) before and (b) after recalibration to local regions........ 9

(a)........ 9

(b) 10

Supplementary Figure 4: Decision curve analysis during internal-external cross validation....... 11

Supplementary Figure 5: Sensitivity, specificity, positive predictive values (PPVs) and negative predictive values (NPVs) of the 4C Deterioration model in the London validation cohort (n= 8,239 participants)........ 12

Supplementary Figure 6: 4C Deterioration vs Mortality predictions for London validation cohort (n = 8,239) shown by (a) sex and (b) ethnicity........ 13

Supplementary Figure 7: Multivariable associations between predictors and outcome using alternative multiple imputation approach as sensitivity analysis........ 14

Supplementary Figure 8: Internal-external cross validation of model by NHS region using alternative multiple imputation approach as sensitivity analysis........ 16

Supplementary Figure 9: Multivariable associations between predictors and outcome using alternative definition of nosocomial infection (>5 days from admission) as sensitivity analysis. 17

Supplementary Figure 10: Multivariable associations between predictors and outcome using alternative definition of nosocomial infection (>10 days from admission) as sensitivity analysis. 19

Supplementary Table 1: Candidate predictors considered during backward variable elimination. 21

Supplementary Table 2: Characteristics of the study cohort, stratified by community vs nosocomial infection. 22

Supplementary Table 3: Final model parameters for 4C Deterioration prognostic model........... 24
Supplementary Table 4: Validation in complete case London data as sensitivity analysis.......... 25
Supplementary Table 5: Validation in London data stratified by time from admission or first COVID-19 assessment to deterioration as sensitivity analysis. 26
Supplementary Table 6: Validation in London data excluding patients still hospitalised at the end of follow-up as sensitivity analysis (n = 7,913 participants). 27
Supplementary Table 7: Validation in London data stratified by (a) community (n = 7,771 participants) vs (b) nosocomial infection (n = 468 participants) as sensitivity analysis. 28
(a).. 28
(b) 28
Supplementary Table 8: Validation parameters of prognostic model among community-acquired cases in London, excluding those with symptom onset recorded after admission date, as sensitivity analysis (n = 7,167 participants). 29
Supplementary Table 9: Validation parameters of prognostic model in London cohort using alternative multiple imputation approach as sensitivity analysis (n = 8,239 participants). 30
Supplementary Table 10: Validation parameters of single continuous predictors included in 4C Deterioration prognostic model in London cohort (n = 8,239 participants). 31
References 32

ISARIC4C Investigators

Consortium Lead Investigator: J Kenneth Baillie, Chief Investigator: Malcolm G Semple, Co-Lead Investigator: Peter JM Openshaw. ISARIC Clinical Coordinator: Gail Carson. Co-Investigators: Beatrice Alex, Benjamin Bach, Wendy S Barclay, Debby Bogaert, Meera Chand, Graham S Cooke, Annemarie B Docherty, Jake Dunning, Ana da Silva Filipe, Tom Fletcher, Christopher A Green, Ewen M Harrison, Julian A Hiscox, Antonia Ying Wai Ho, Peter W Horby, Samreen Ijaz, Saye Khoo, Paul Klenerman, Andrew Law, Wei Shen Lim, Alexander J Mentzer, Laura Merson, Alison M Meynert, Mahdad Noursadeghi, Shona C Moore, Massimo Palmarini, William A Paxton, Georgios Pollakis, Nicholas Price, Andrew Rambaut, David L Robertson, Clark D Russell, Vanessa Sancho-Shimizu, Janet T Scott, Thushan de Silva, Louise Sigfrid, Tom Solomon, Shiranee Sriskandan, David Stuart, Charlotte Summers, Richard S Tedder, Emma C Thomson, AA Roger Thompson, Ryan S Thwaites, Lance CW Turtle, Maria Zambon. Project Managers: Hayley Hardwick, Chloe Donohue, Ruth Lyons, Fiona Griffiths, Wilna Oosthuyzen. Data Analysts: Lisa Norman, Riinu Pius, Tom M Drake, Cameron J Fairfield, Stephen Knight, Kenneth A Mclean, Derek Murphy, Catherine A Shaw. Data and Information System Managers: Jo Dalton, James Lee, Daniel Plotkin, Michelle Girvan, Scott Mullaney, Claire Petersen, Egle Saviciute, Stephanie Roberts, Janet Harrison, Laura Marsh, Marie Connor, Sophie Halpin, Clare Jackson, Carrol Gamble. Data integration and presentation: Gary Leeming, Andrew Law, Murray Wham, Sara Clohisey, Ross Hendry, James Scott-Brown. Material Management: William Greenhalf, Victoria Shaw, Sarah McDonald. Patient engagement: Seán Keating. Outbreak Laboratory Staff and Volunteers: Katie A. Ahmed, Jane A Armstrong, Milton Ashworth, Innocent G Asiimwe, Siddharth Bakshi, Samantha L Barlow, Laura Booth, Benjamin Brennan, Katie Bullock, Benjamin WA Catterall, Jordan J Clark, Emily A Clarke, Sarah Cole, Louise Cooper, Helen Cox, Christopher Davis, Oslem Dincarslan, Chris Dunn, Philip Dyer, Angela Elliott, Anthony Evans, Lorna Finch, Lewis WS Fisher, Terry Foster, Isabel Garcia-Dorival, Willliam Greenhalf, Philip Gunning, Catherine Hartley, Antonia Ho, Rebecca L Jensen, Christopher B Jones, Trevor R Jones, Shadia Khandaker, Katharine King, Robyn T. Kiy, Chrysa Koukorava, Annette Lake, Suzannah Lant, Diane Latawiec, L Lavelle-Langham, Daniella Lefteri, Lauren Lett, Lucia A Livoti, Maria Mancini, Sarah McDonald, Laurence McEvoy, John McLauchlan, Soeren Metelmann, Nahida S Miah, Joanna Middleton, Joyce Mitchell, Shona C Moore, Ellen G Murphy, Rebekah Penrice-Randal, Jack Pilgrim, Tessa Prince, Will Reynolds, P. Matthew Ridley, Debby Sales, Victoria E Shaw, Rebecca K Shears, Benjamin Small, Krishanthi S Subramaniam, Agnieska Szemiel, Aislynn Taggart, Jolanta Tanianis-Hughes, Jordan Thomas, Erwan Trochu, Libby van Tonder, Eve Wilcock, J. Eunice Zhang. Local Principal Investigators: Kayode Adeniji, Daniel Agranoff, Ken Agwuh, Dhiraj Ail, Ana Alegria, Brian Angus, Abdul Ashish, Dougal Atkinson, Shahedal

Bari, Gavin Barlow, Stella Barnass, Nicholas Barrett, Christopher Bassford, David Baxter, Michael Beadsworth, Jolanta Bernatoniene, John Berridge, Nicola Best, Pieter Bothma, David Brealey, Robin Brittain-Long, Naomi Bulteel, Tom Burden, Andrew Burtenshaw, Vikki Caruth, David Chadwick, Duncan Chambler, Nigel Chee, Jenny Child, Srikanth Chukkambotla, Tom Clark, Paul Collini, Catherine Cosgrove, Jason Cupitt, Maria-Teresa Cutino-Moguel, Paul Dark, Chris Dawson, Samir Dervisevic, Phil Donnison, Sam Douthwaite, Ingrid DuRand, Ahilanadan Dushianthan, Tristan Dyer, Cariad Evans, Chi Eziefula, Chrisopher Fegan, Adam Finn, Duncan Fullerton, Sanjeev Garg, Sanjeev Garg, Atul Garg, Effrossyni Gkrania-Klotsas, Jo Godden, Arthur Goldsmith, Clive Graham, Elaine Hardy, Stuart Hartshorn, Daniel Harvey, Peter Havalda, Daniel B Hawcutt, Maria Hobrok, Luke Hodgson, Anil Hormis, Michael Jacobs, Susan Jain, Paul Jennings, Agilan Kaliappan, Vidya Kasipandian, Stephen Kegg, Michael Kelsey, Jason Kendall, Caroline Kerrison, Ian Kerslake, Oliver Koch, Gouri Koduri, George Koshy, Shondipon Laha, Steven Laird, Susan Larkin, Tamas Leiner, Patrick Lillie, James Limb, Vanessa Linnett, Jeff Little, Michael MacMahon, Emily MacNaughton, Ravish Mankregod, Huw Masson, Elijah Matovu, Katherine McCullough, Ruth McEwen, Manjula Meda, Gary Mills, Jane Minton, Mariyam Mirfenderesky, Kavya Mohandas, Quen Mok, James Moon, Elinoor Moore, Patrick Morgan, Craig Morris, Katherine Mortimore, Samuel Moses, Mbiye Mpenge, Rohinton Mulla, Michael Murphy, Megan Nagel, Thapas Nagarajan, Mark Nelson, Igor Otahal, Mark Pais, Selva Panchatsharam, Hassan Paraiso, Brij Patel, Natalie Pattison, Justin Pepperell, Mark Peters, Mandeep Phull, Stefania Pintus, Jagtur Singh Pooni, Frank Post, David Price, Rachel Prout, Nikolas Rae, Henrik Reschreiter, Tim Reynolds, Neil Richardson, Mark Roberts, Devender Roberts, Alistair Rose, Guy Rousseau, Brendan Ryan, Taranprit Saluja, Aarti Shah, Prad Shanmuga, Anil Sharma, Anna Shawcross, Jeremy Sizer, Manu Shankar-Hari, Richard Smith, Catherine Snelson, Nick Spittle, Nikki Staines, Tom Stambach, Richard Stewart, Pradeep Subudhi, Tamas Szakmany, Kate Tatham, Jo Thomas, Chris Thompson, Robert Thompson, Ascanio Tridente, Darell Tupper-Carey, Mary Twagira, Andrew Ustianowski, Nick Vallotton, Lisa Vincent-Smith, Shico Visuvanathan, Alan Vuylsteke, Sam Waddy, Rachel Wake, Andrew Walden, Ingeborg Welters, Tony Whitehouse, Paul Whittaker, Ashley Whittington, Meme Wijesinghe, Martin Williams, Lawrence Wilson, Sarah Wilson, Stephen Winchester, Martin Wiselka, Adam Wolverson, Daniel G Wooton, Andrew Workman, Bryan Yates, and Peter Young.

Supplementary Methods

Variable selection

We used a logistic regression modelling approach and performed backward elimination of the *a priori* candidate variables using Akaike information criteria (AIC). For continuous variables, linear and spline terms were grouped together and considered collectively for inclusion/exclusion in the model. Variables that led to a reduction in AIC were retained (equivalent to p=0.157 for predictors with 1 degree of freedom), using the fastbw command in the rms package in R[1]. This process was done separately in each multiply imputed dataset and in each NHS region in the development set. Predictors were required to be retained in >50% of multiply imputed datasets in >50% of development NHS regions in order to enter the final model. We specified this in order to retain a parsimonious set of predictors that had consistent prognostic value across the development NHS regions.

Missing data

We handled missing data (including predictors and outcomes) using multiple imputation with chained equations, assuming missingness at random[2], using the mice package in R[3]. We included all predictors (including restricted cubic spline transformations) and the outcome in the imputation models to ensure compatibility. We used predictive mean matching for continuous variables, logistic regression imputation for binary data, polytomous regression imputation for unordered categorical data and proportional odds model for ordered categorical data[3]. We assessed convergence of the imputation models by examining convergence plots and examined the distributions of imputed values to ensure plausibility. Imputation was done separately for each NHS region to preserve potential inter-region heterogeneity. We generated 10 multiply imputed datasets; all primary analyses were performed in each imputed dataset and model and validation parameters were pooled using Rubin's rules[4].

Validation metrics

C-statistics were calculated using the pROC package in R[5]. Calibration slopes were calculated by fitting a logistic regression model for the outcome, with the model linear predictor as the sole independent variable; the coefficient for the linear predictor provides the calibration slope metric. Calibration-in-the-large was computed by fitting a logistic regression model for the outcome, with the model linear predictor as an offset term; the intercept of the fitted model provides the calibration-in-the-large. Calibration plots were generated by fitting a loess smoother between model predictions and the outcome in stacked multiply imputed datasets. Recalibration to each region was performed by re-estimating the model intercept in the validation sets during each internal-external cross-validation cycle.

Software

The analyses were conducted in R (version 3.6.3) using tidyverse (version 1.3.0)[6] for data processing, rms (version 6.0-1)[1] for logistic regression modelling, mice (version 3.11.0)[3] for multiple imputation, pROC (version 1.16.2)[5] for C-statistic calculations,rmda (version 1.6)[7] for decision curve analysis and runway (version 0.0.0.9000)[8] for visualising model performance metrics across probability thresholds.

Sample size

We assumed *a priori* that at least 30% of hospital admission would reach the primary outcome. The most comparable existing tools for risk stratification are the qSOFA, NEWS2, and CURB-65 scores[9–11]. qSOFA achieved C-statistics ranging from 0.71-0.78 during external validation[9]. Based on a conservative C-statistic of 0.71, we assumed a Cox-Snell R^2 of 0.24. With 40 candidate predictor parameters (including transformations), the minimum sample size required for new model development was estimated to be 1,290, with 387 events[12,13].

Supplementary Results

Supplementary Figure 1: Days to deterioration following admission, stratified by first chronological type of deterioration.

Outcome events are shown as stacked histogram. Total sample size = 74,944 participants with 30,899 deterioration events with known time point shown in plot. Median days to deterioration was 4 (interquartile range 1-9). Noninvasive = non-invasive ventilation; ICU = high dependency or intensive care unit admission; IMV = invasive mechanical ventilation; ECMO = extra-corporeal membrane oxygenation.

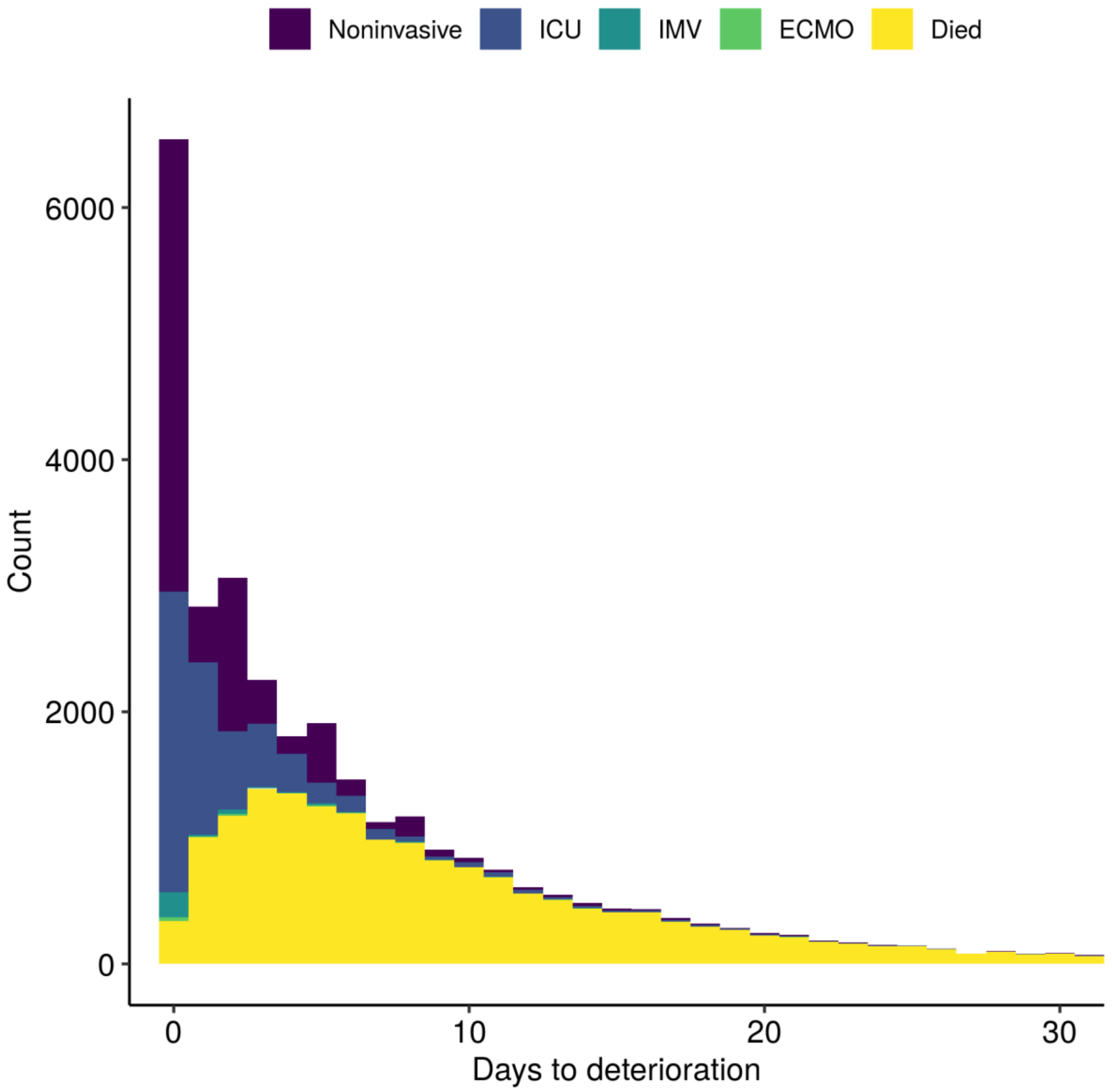

Supplementary Figure 2: Heatmap showing missingness (%) of candidate predictors and outcome, stratified by NHS region (n = 74,944 participants).

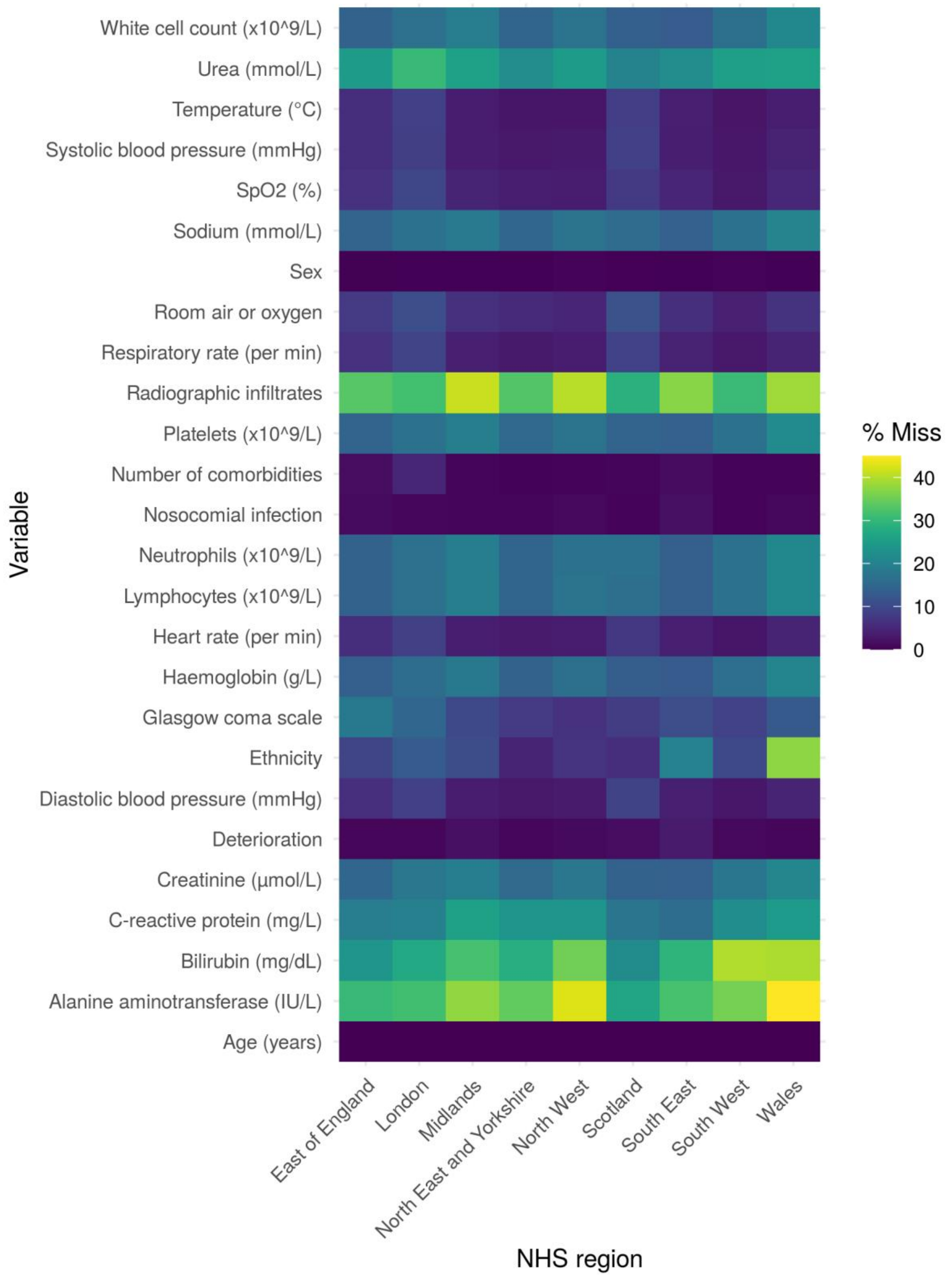

Supplementary Figure 3: Pooled calibration across multiple imputed datasets during IECV (a) before and (b) after recalibration to local regions.

Calibration is shown using a loess-smoother across multiply imputed datasets. Rug plots indicate the distributions of predicted risk. Total sample size = 66,705 participants.

(a)

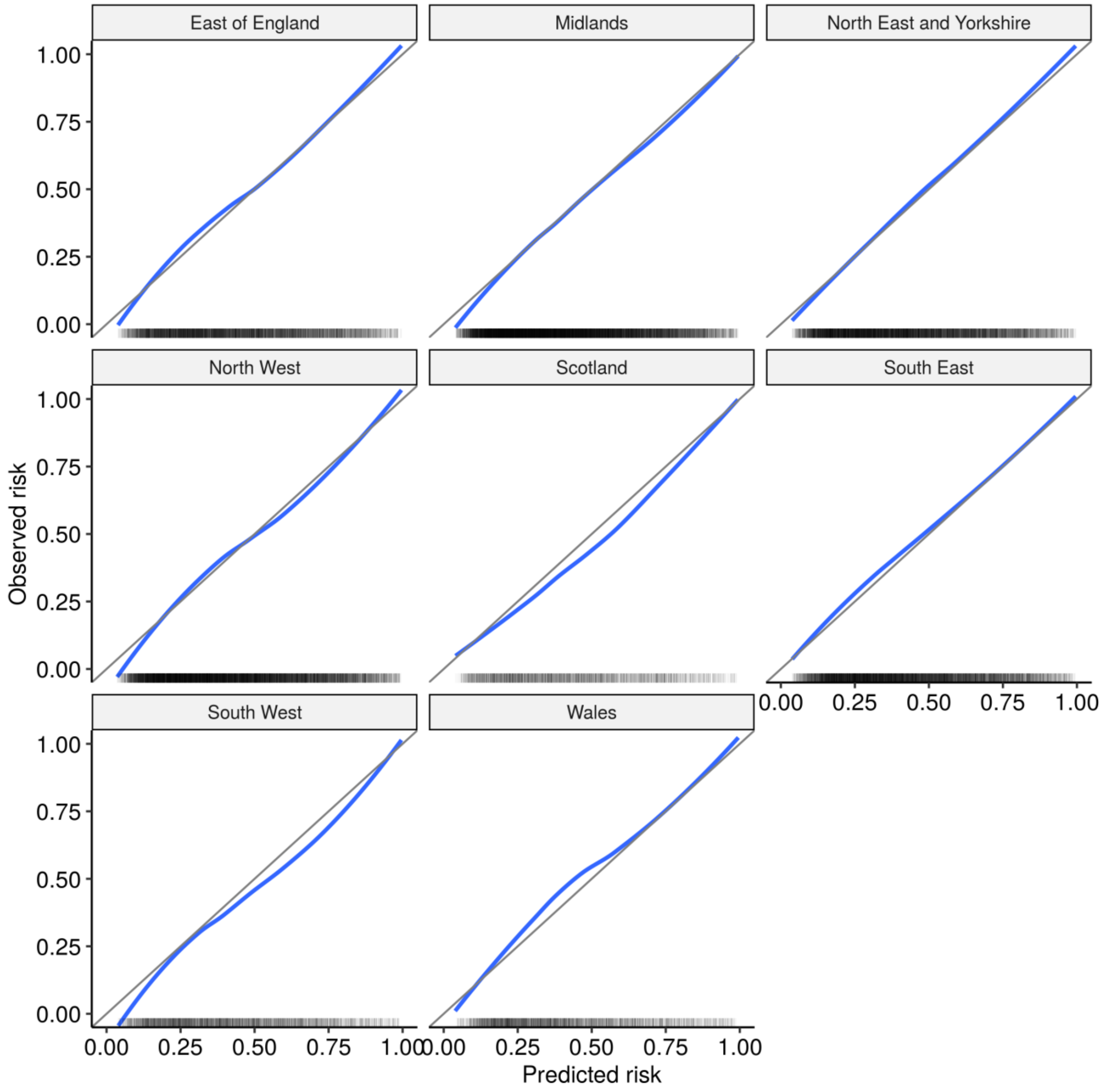

(b)

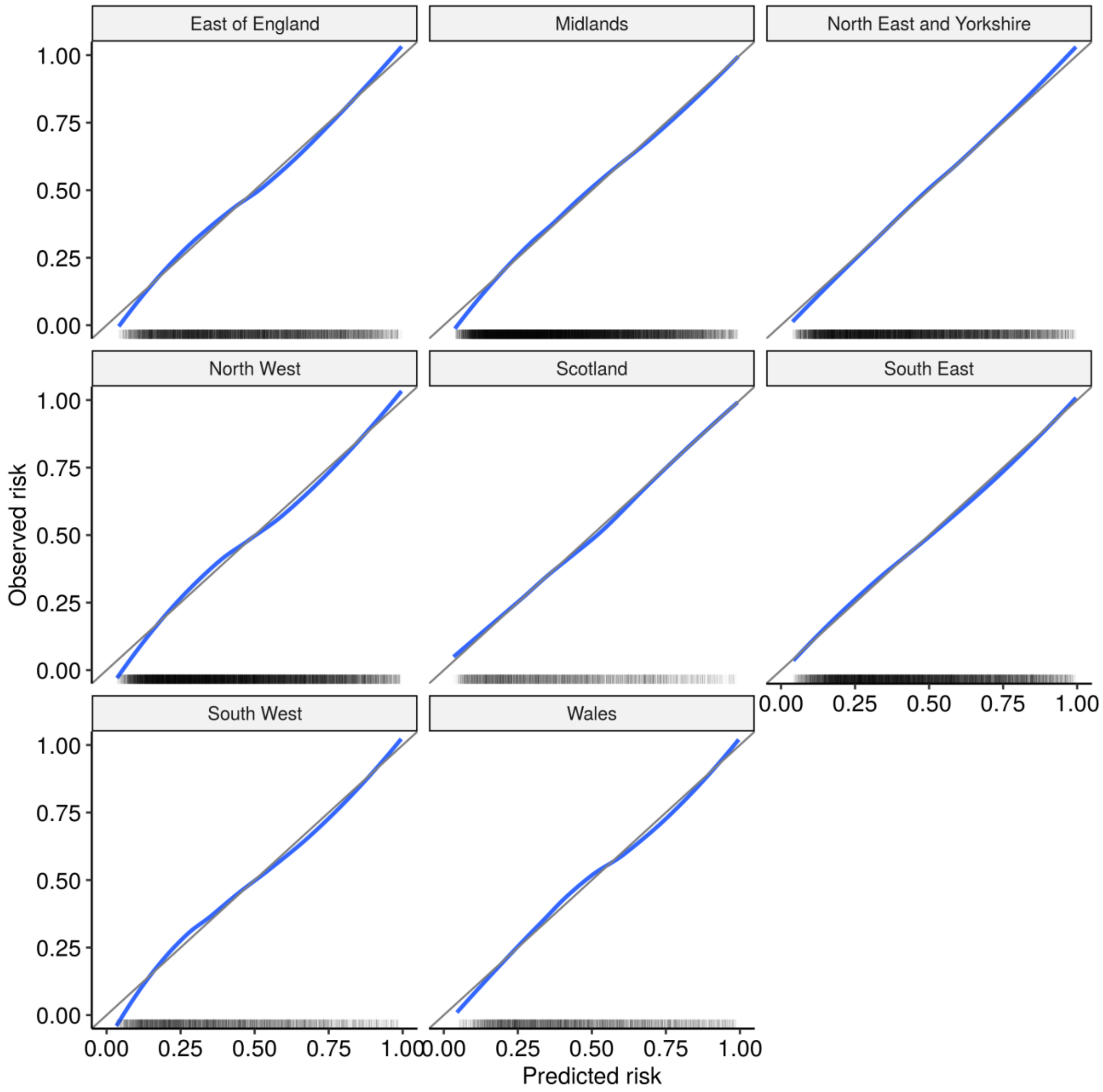
East of England
Midlands
North East and Yorkshire
North West
Scotland
South East
South West
Wales
Observed risk
Predicted risk
0.00
0.25
0.50
0.75
1.00

Supplementary Figure 4: Decision curve analysis during internal-external cross validation.

Shown for the 4C Deterioration model without recalibration to local region. Net benefit is shown for each candidate model compared to the ‘treat all’ and ‘treat none’ approaches. Points score models are recalibrated to the validation data, resulting in optimistic estimates of net benefit for these models. Total sample size = 66,705 participants.

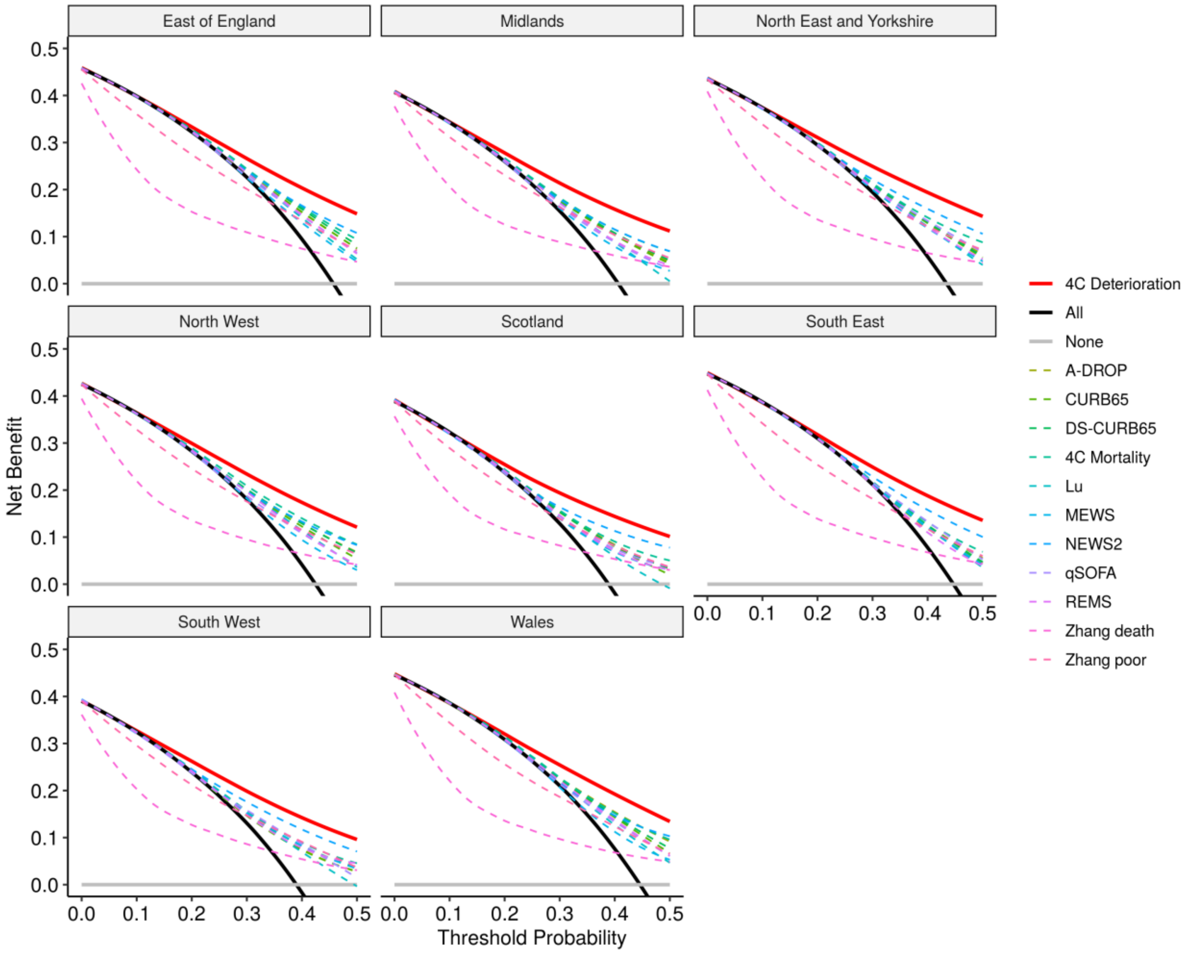

Supplementary Figure 5: Sensitivity, specificity, positive predictive values (PPVs) and negative predictive values (NPVs) of the 4C Deterioration model in the London validation cohort (n= 8,239 participants).

Each metric is shown across the full range of probability thresholds for the model predictions for illustration. The distribution of predictions is shown in the histogram. Positive predictive value at a threshold of 0% and negative predictive value at a threshold of 100% reflect prior probability due to the overall observed risk of deterioration in the validation cohort.

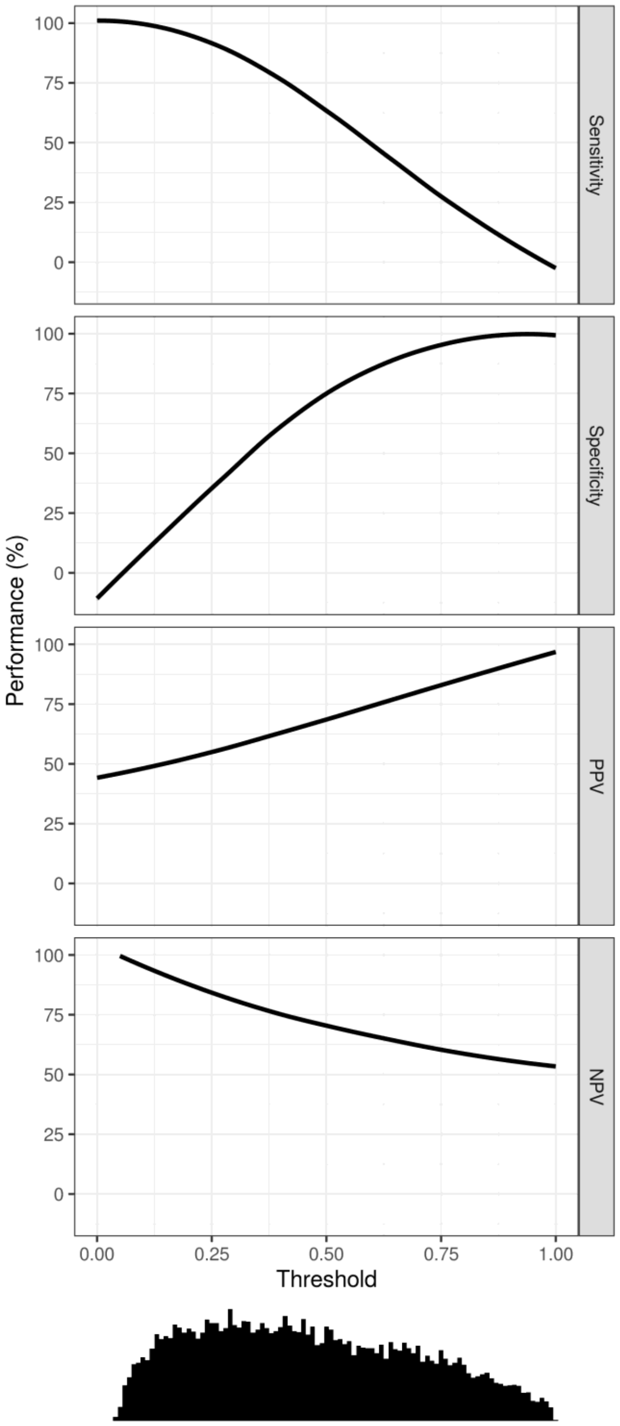

Supplementary Figure 6: 4C Deterioration vs Mortality predictions for London validation cohort (n = 8,239) shown by (a) sex and (b) ethnicity.

Both plots are shown stratified by age in years. 4C Mortality probabilities are calculated from points scores, based on observed mortality risk for each score in the original validation data. Smoothed lines reflects loess fit.

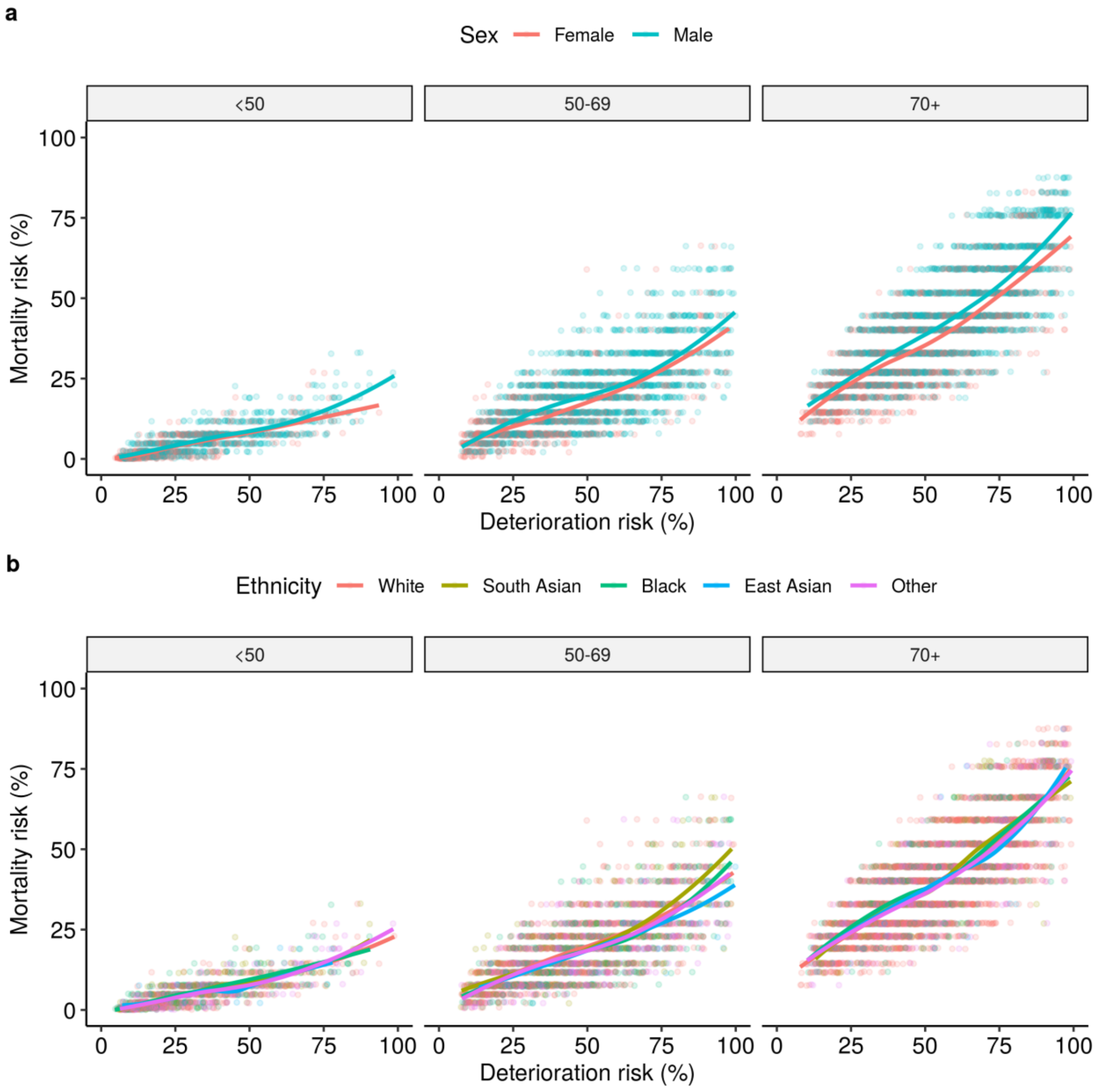

Supplementary Figure 7: Multivariable associations between predictors and outcome using alternative multiple imputation approach as sensitivity analysis.

Total development sample size = 66,705 participants. Black lines and dots indicate point estimates; grey shaded regions and error bars indicate 95% confidence intervals.

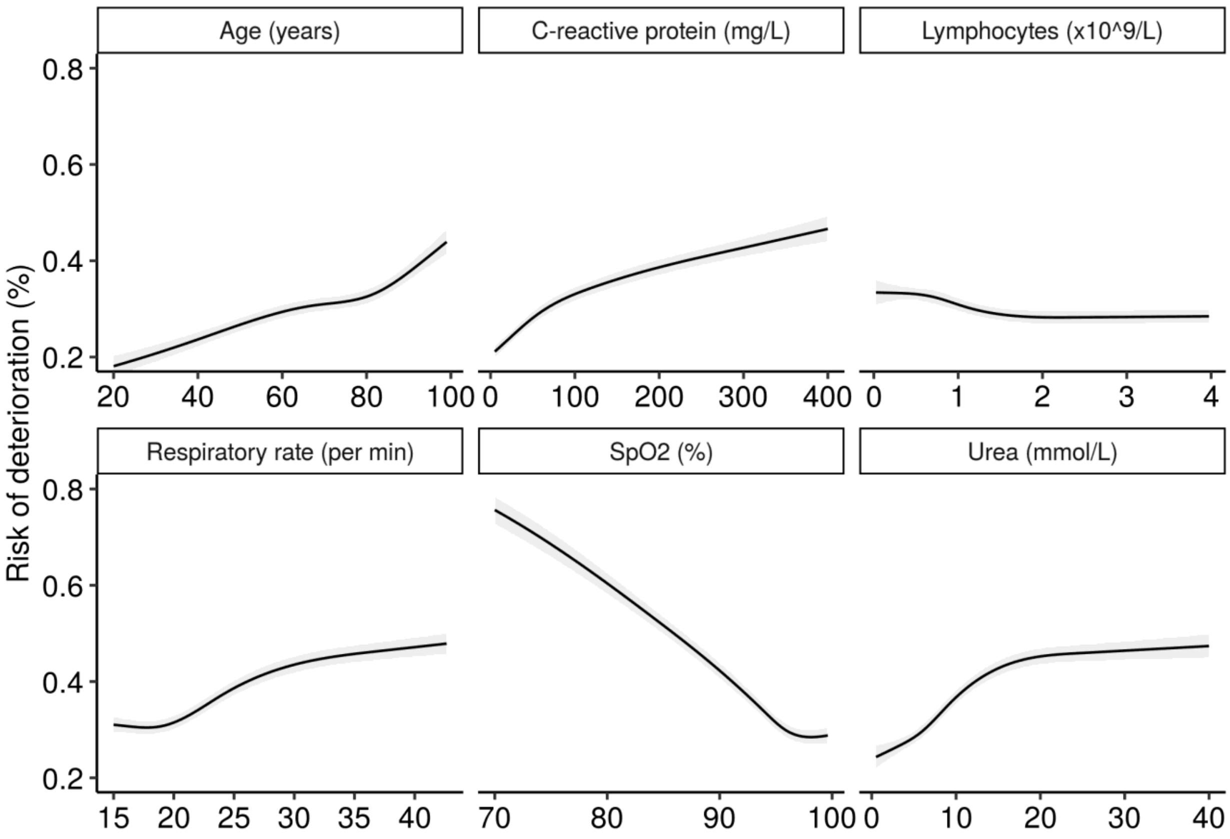
Age (years)
C-reactive protein (mg/L)
Lymphocytes (x10^9/L)
Respiratory rate (per min)
SpO2 (%)
Urea (mmol/L)
Risk of deterioration (%)
0.8
0.6
0.4
0.2
20 40 60 80 100
0 100 200 300 400
0 1 2 3 4
15 20 25 30 35 40
70 80 90 100
0 10 20 30 40

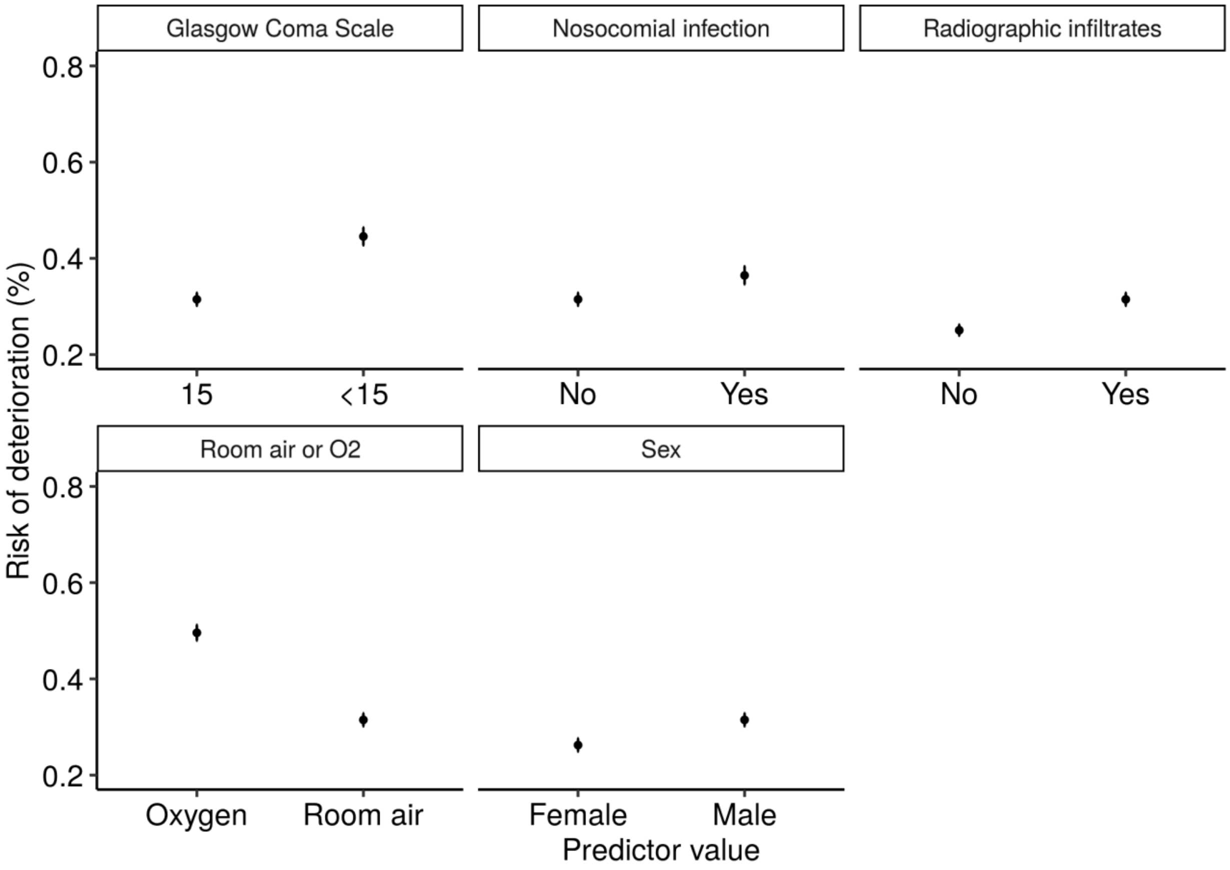
Glasgow Coma Scale
Nosocomial infection
Radiographic infiltrates
Room air or O2
Sex
Risk of deterioration (%)
0.8
0.6
0.4
0.2
15
<15
No
Yes
No
Yes
Oxygen
Room air
Female
Male
Predictor value

Supplementary Figure 8: Internal-external cross validation of model by NHS region using alternative multiple imputation approach as sensitivity analysis.

Pooled estimates are calculated through random-effects meta-analysis. Total sample size = 66,705 participants. Black squares indicate point estimates; bars indicate 95% confidence intervals; diamonds indicate pooled random-effects meta-analysis estimates.

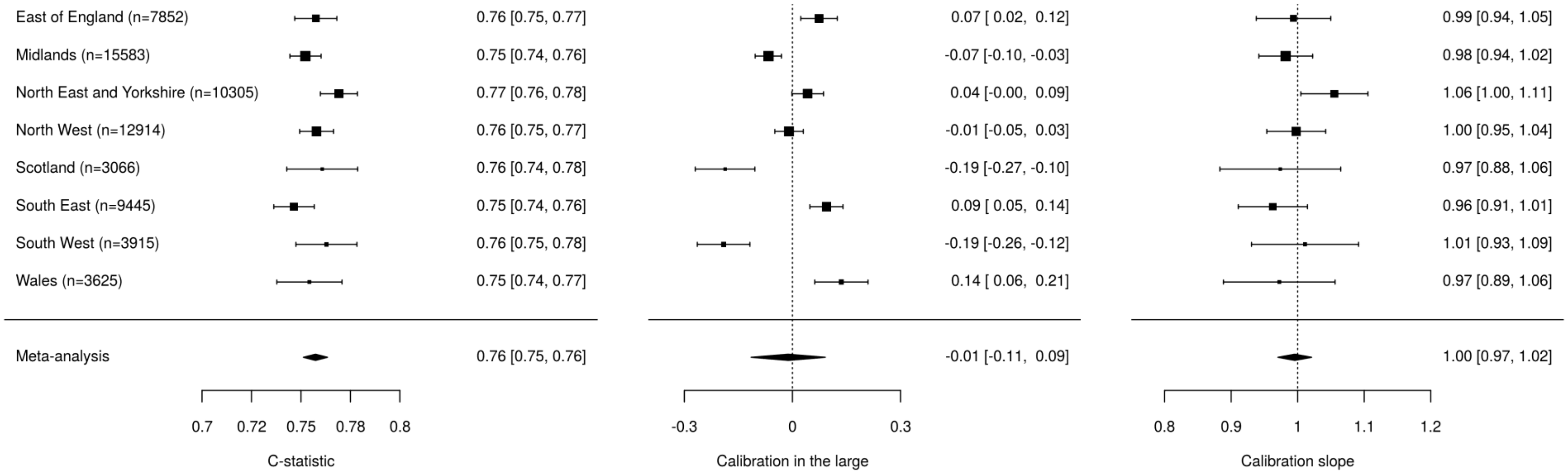

Supplementary Figure 9: Multivariable associations between predictors and outcome using alternative definition of nosocomial infection (>5 days from admission) as sensitivity analysis.

Total development sample size = 66,705 participants. Black lines and dots indicate point estimates; grey shaded regions and error bars indicate 95% confidence intervals.

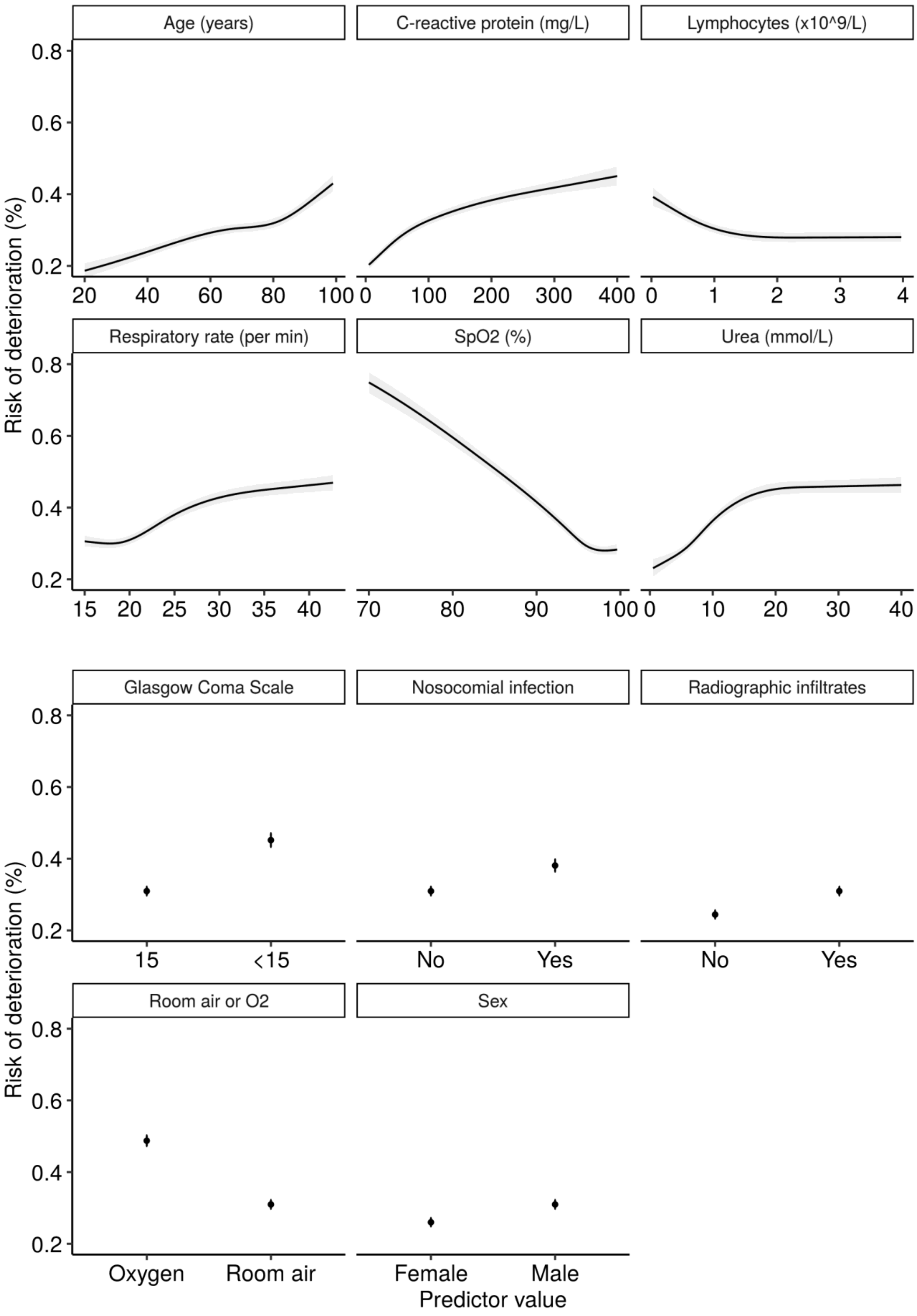
Age (years)
C-reactive protein (mg/L)
Lymphocytes (x10^9/L)
Respiratory rate (per min)
SpO2 (%)
Urea (mmol/L)
Glasgow Coma Scale
Nosocomial infection
Radiographic infiltrates
Room air or O2
Sex
Risk of deterioration (%)
Predictor value
15
<15
No
Yes
Oxygen
Room air
Female
Male

Supplementary Figure 10: Multivariable associations between predictors and outcome using alternative definition of nosocomial infection (>10 days from admission) as sensitivity analysis.

Total development sample size = 66,705 participants. Black lines and dots indicate point estimates; grey shaded regions and error bars indicate 95% confidence intervals.

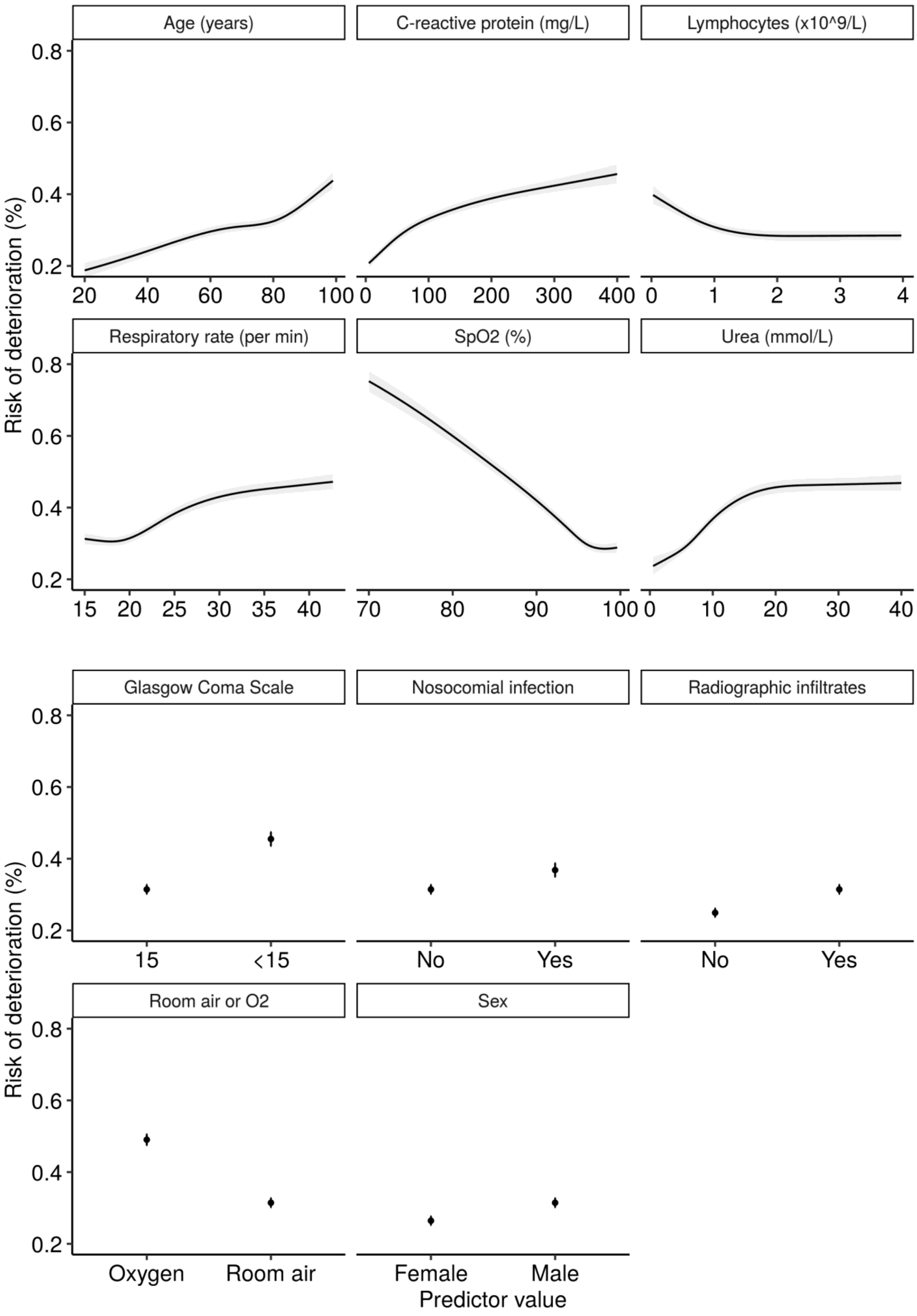
Age (years)
C-reactive protein (mg/L)
Lymphocytes (x10^9/L)
Respiratory rate (per min)
SpO2 (%)
Urea (mmol/L)
Risk of deterioration (%)
0.2
0.4
0.6
0.8
20 40 60 80 100
0 100 200 300 400
0 1 2 3 4
15 20 25 30 35 40
70 80 90 100
0 10 20 30 40
Glasgow Coma Scale
Nosocomial infection
Radiographic infiltrates
Room air or O2
Sex
15
<15
No
Yes
Oxygen
Room air
Female
Male
Predictor value

Supplementary Table 1: Candidate predictors considered during backward variable elimination.

Candidate predictor	Type	Levels for factor variables
Patient demographics		
Age on admission (years)	Numeric	
Sex at Birth	Factor	Male, Female
Ethnicity	Factor	White, Black, East Asian, South Asian, or Other
Number of comorbidities*	Numeric	
Nosocomial infection**	Factor	Yes, No
Clinical signs / observations		
Respiratory rate (per min)	Numeric	
Peripheral oxygen saturation (%)	Numeric	
Breathing room air or oxygen	Factor	Room air, Oxygen
Systolic blood pressure (mmHg)	Numeric	
Diastolic blood pressure (mmHg)	Numeric	
Temperature (<b0>C)	Numeric	
Heart Rate (per min)	Numeric	
Glasgow Coma Scale	Factor	15, <15
Laboratory measures		
Haemoglobin (g/L)	Numeric	
White cell count (x10^9/L)	Numeric	
Neutrophil count (x10^9/L)	Numeric	
Lymphocyte count (x10^9/L)	Numeric	
Platelet Count (x10^9/L)	Numeric	
Sodium (mmol/L)	Numeric	
Total Bilirubin (mg/dL)	Numeric	
Alanine aminotransferase (IU/L)	Numeric	
Urea (mmol/L)	Numeric	
Creatinine (<b5>mol/L)	Numeric	
C-reactive protein (mg/L)	Numeric	
Radiology		
Infiltrates on chest radiograph	Factor	Yes, No

All variables were taken from point of hospital admission, or the day of first clinical assessment for COVID-19.,

*Comorbidities included chronic cardiac disease, chronic respiratory disease (excluding asthma), chronic renal disease, mild to severe liver disease, dementia, chronic neurological disease, connective tissue disease, diabetes mellitus, HIV or AIDS, malignancy and clinician-defined obesity.

**Community-acquired infection was defined as symptom onset or first positive SARS-CoV-2 PCR within 7 days from admission; participants who did not meet these criteria and had either symptom onset or first positive SARS-CoV-2 PCR >7 days from admission were classified as nosocomial cases.

Supplementary Table 2: Characteristics of the study cohort, stratified by community vs nosocomial infection.

Characteristic	Overall, N = 74,944	Community, N = 66,980[1]	Nosocomial, N = 7,320[1]	(Missing), N = 644[1]
Age (years)	75 (60, 84)	74 (59, 84)	80 (69, 87)	78 (64, 87)
Sex				
Female	32,807 (44%)	29,052 (43%)	3,445 (47%)	310 (48%)
Male	41,993 (56%)	37,809 (57%)	3,851 (53%)	333 (52%)
Unknown	144	119	24	1
Ethnicity				
White	55,016 (83%)	48,706 (82%)	5,843 (91%)	467 (89%)
South Asian	3,520 (5.3%)	3,356 (5.6%)	150 (2.3%)	14 (2.7%)
Black	2,553 (3.8%)	2,432 (4.1%)	106 (1.7%)	15 (2.9%)
East Asian	492 (0.7%)	474 (0.8%)	17 (0.3%)	1 (0.2%)
Other	4,844 (7.3%)	4,514 (7.6%)	303 (4.7%)	27 (5.2%)
Unknown	8,519	7,498	901	120
SARS-CoV-2 PCR positive	66,136 (97%)	59,356 (97%)	6,769 (99%)	11 (9.3%)
Unknown	6,715	5,676	513	526
Number of comorbidities	1 (1, 2)	1 (1, 2)	2 (1, 3)	1 (1, 2)
Unknown	839	655	58	126
Radiographic infiltrates	29,579 (62%)	28,458 (63%)	1,032 (48%)	89 (39%)
Unknown	27,195	21,599	5,178	418
Temperature (°C)	37.2 (36.5, 38.1)	37.2 (36.6, 38.1)	36.8 (36.4, 37.6)	36.7 (36.3, 37.1)
Unknown	3,106	2,365	579	162
Heart rate (per min)	90 (78, 104)	90 (78, 105)	85 (72, 98)	86 (74, 98)
Unknown	3,383	2,533	681	169
Respiratory rate (per min)	20 (18, 26)	22 (18, 26)	18 (17, 20)	18 (17, 20)
Unknown	3,535	2,585	787	163
Systolic blood pressure (mmHg)	130 (114, 147)	130 (114, 146)	130 (113, 148)	131 (115, 152)
Unknown	3,187	2,356	672	159
Diastolic blood pressure (mmHg)	74 (64, 84)	74 (65, 84)	72 (62, 82)	74 (65, 84)
Unknown	3,330	2,482	688	160
SpO2 (%)	95 (92, 97)	95 (92, 97)	96 (94, 98)	96 (95, 98)
Unknown	3,756	2,869	724	163
Room air or oxygen				
Room air	48,574 (69%)	42,455 (67%)	5,716 (88%)	403 (87%)
Oxygen	21,453 (31%)	20,588 (33%)	804 (12%)	61 (13%)
Unknown	4,917	3,937	800	180
Glasgow coma scale	15 (15, 15)	15 (15, 15)	15 (15, 15)	15 (15, 15)
Unknown	7,839	6,690	932	217
Haemoglobin (g/L)	128 (112, 142)	129 (113, 143)	111 (96, 128)	120 (104, 135)
Unknown	11,748	8,417	3,145	186
White cell count (x10^9/L)	7.5 (5.4, 10.7)	7.6 (5.5, 10.7)	7.2 (5.2, 10.2)	8.4 (6.0, 11.3)
Unknown	12,130	8,768	3,173	189
Lymphocytes (x10^9/L)	0.90 (0.60, 1.30)	0.90 (0.60, 1.30)	0.90 (0.60, 1.30)	1.00 (0.60, 1.40)
Unknown	12,345	8,944	3,211	190
Neutrophils (x10^9/L)	5.8 (3.9, 8.7)	5.8 (3.9, 8.7)	5.3 (3.5, 8.1)	6.5 (4.3, 9.3)
Unknown	12,308	8,923	3,197	188
Platelets (x10^9/L)	221 (167, 290)	219 (166, 287)	245 (178, 331)	242 (180, 312)
Unknown	12,463	9,066	3,208	189
Alanine aminotransferase (IU/L)	25 (16, 43)	25 (16, 43)	21 (14, 37)	20 (13, 39)
Unknown	26,735	21,399	5,024	312
Bilirubin (mg/dL)	10 (7, 14)	10 (7, 14)	8 (5, 13)	10 (7, 16)
Unknown	22,931	17,779	4,843	309

Characteristic	Overall, N = 74,944	Community, N = 66,980[1]	Nosocomial, N = 7,320[1]	(Missing), N = 644[1]
Urea (mmol/L)	7 (5, 11)	7 (5, 11)	7 (5, 11)	8 (5, 12)
Unknown	18,509	14,642	3,624	243
Creatinine (µmol/L)	86 (67, 121)	86 (67, 121)	81 (61, 116)	89 (66, 123)
Unknown	12,656	9,276	3,185	195
Sodium (mmol/L)	137 (134, 140)	137 (134, 140)	137 (134, 141)	137 (134, 141)
Unknown	12,277	8,939	3,143	195
C-reactive protein (mg/L)	80 (33, 154)	82 (34, 157)	53 (19, 106)	52 (13, 118)
Unknown	16,318	12,548	3,534	236
NHS region				
East of England	7,852 (10%)	7,188 (11%)	564 (7.7%)	100 (16%)
London	8,239 (11%)	7,721 (12%)	465 (6.4%)	53 (8.2%)
Midlands	15,583 (21%)	14,057 (21%)	1,446 (20%)	80 (12%)
North East and Yorkshire	10,305 (14%)	9,391 (14%)	857 (12%)	57 (8.9%)
North West	12,914 (17%)	11,135 (17%)	1,652 (23%)	127 (20%)
Scotland	3,066 (4.1%)	2,811 (4.2%)	242 (3.3%)	13 (2.0%)
South East	9,445 (13%)	8,310 (12%)	969 (13%)	166 (26%)
South West	3,915 (5.2%)	3,495 (5.2%)	403 (5.5%)	17 (2.6%)
Wales	3,625 (4.8%)	2,872 (4.3%)	722 (9.9%)	31 (4.8%)
Deterioration				
Ventilatory support or HDU/ICU	15,039 (20%)	14,434 (22%)	541 (7.4%)	64 (9.9%)
Died	16,885 (23%)	14,691 (22%)	2,093 (29%)	101 (16%)
No deterioration	42,024 (56%)	37,131 (55%)	4,542 (62%)	351 (55%)
(Missing)	996 (1.3%)	724 (1.1%)	144 (2.0%)	128 (20%)
Days from admission to COVID-19 assessment	0 (0, 0)	0 (0, 0)	18 (11, 35)	0 (0, 2)
Unknown	1,767	1,611	96	60

[1]Statistics presented: median (IQR); n (%)

Supplementary Table 3: Final model parameters for 4C Deterioration prognostic model.

Parameters are pooled across multiply imputed datasets. Total development sample size = 66,705 participants.

Characteristic	log(OR)[1]
Intercept	4.033
Age (years)	0.0159
Age (spline 1)	-0.0129
Age (spline 2)	0.1265
Sex	
Female	—
Male	0.2690
Nosocomial	
No	—
Yes	0.2439
Radiographic infiltrates	
No	—
Yes	0.3252
Respiratory rate (per min)	-0.0145
Respiratory rate (spline 1)	0.5992
Respiratory rate (spline 2)	-1.078
SpO2 (%)	-0.0707
SpO2 (spline 1)	-0.0248
SpO2 (spline 2)	1.024
Room air or oxygen	
Room air	—
Oxygen therapy	0.7450
Glasgow Coma Scale	
15	—
<15	0.6028
Urea (mmol/L)	0.0508
Urea (spline 1)	0.4446
Urea (spline 2)	-1.035
C-reactive protein (mg/L)	0.0097
C-reactive protein (spline 1)	-0.0395
C-reactive protein (spline 2)	0.0588
Lymphocytes (x10^9/L)	-0.4564
Lymphocytes (spline 1)	0.7309
Lymphocytes (spline 2)	-0.8113

[1]OR = Odds Ratio
Restricted cubic spline knot positions are:
- Age = 38.5, 67.7, 81.1, 92.9;
- Respiratory rate = 16, 19, 24, 37;
- SpO2 = 84, 94, 96, 100;
- Urea = 2.9, 5.7, 9.2, 25.5;
- C-reactive protein = 5, 45, 113, 297;
- Lymphocytes = 0.3, 0.7, 1.1, 2.4.

Supplementary Table 4: Validation in complete case London data as sensitivity analysis.

Models are shown for prediction of in-hospital clinical deterioration and are sorted by C-statistic. CITL = calibration-in-the-large. 'Original outcome' column indicates original intended outcome for each candidate model during development. CITL and slopes are not shown for points score models since they are not on probability scale.

Score	Original outcome	n	C-statistic	CITL	Slope
4C Deterioration	Deterioration (in-hospital)	3766	0.76 (0.75 - 0.78)	-0.06 (-0.14 - 0.01)	0.96 (0.89 - 1.04)
NEWS2	Deterioration (1 day)	6410	0.69 (0.67 - 0.7)		
Zhang "death"	Mortality (in-hospital)	6290	0.68 (0.67 - 0.69)	2.33 (2.26 - 2.39)	0.15 (0.13 - 0.17)
Zhang "poor"	Deterioration (in-hospital)	6290	0.68 (0.66 - 0.69)	0.63 (0.56 - 0.69)	0.13 (0.11 - 0.15)
4C Mortality	Mortality (in-hospital)	4805	0.67 (0.66 - 0.69)		
REMS	Mortality (in-hospital)	6503	0.66 (0.65 - 0.67)		
DS-CURB65	Mortality (30 days)	4825	0.66 (0.64 - 0.67)		
A-DROP	Mortality (30 days)	5043	0.64 (0.63 - 0.66)		
CURB65	Mortality (30 days)	5073	0.64 (0.63 - 0.66)		
qSOFA	Mortality (in-hospital)	6742	0.63 (0.62 - 0.64)		
MEWS	Deterioration (in-hospital)	6564	0.62 (0.61 - 0.64)		
Lu	Mortality (12 days)	6619	0.61 (0.6 - 0.62)		

Supplementary Table 5: Validation in London data stratified by time from admission or first COVID-19 assessment to deterioration as sensitivity analysis.

C-statistics (with 95% confidence intervals) are shown for each model for prediction of in-hospital clinical deterioration, and stratified by time to deterioration as indicated. 'Original outcome' column indicates original intended outcome for each candidate model during development.

Score	Original outcome	Deterioration on day 0	Deterioration after day 0	Deterioration 0 to 3 days	Deterioration after 3 days
4C Deterioration	Deterioration (in-hospital)	0.82 (0.8 - 0.83)	0.75 (0.74 - 0.76)	0.8 (0.79 - 0.81)	0.73 (0.71 - 0.74)
4C Mortality	Mortality (in-hospital)	0.62 (0.61 - 0.64)	0.71 (0.7 - 0.72)	0.64 (0.63 - 0.66)	0.74 (0.73 - 0.76)
DS-CURB65	Mortality (30 days)	0.63 (0.61 - 0.64)	0.68 (0.67 - 0.69)	0.63 (0.62 - 0.65)	0.71 (0.7 - 0.73)
REMS	Mortality (in-hospital)	0.66 (0.64 - 0.68)	0.67 (0.66 - 0.69)	0.65 (0.64 - 0.67)	0.69 (0.67 - 0.7)
A-DROP	Mortality (30 days)	0.61 (0.59 - 0.63)	0.67 (0.66 - 0.68)	0.62 (0.6 - 0.63)	0.7 (0.69 - 0.72)
CURB65	Mortality (30 days)	0.63 (0.61 - 0.64)	0.67 (0.65 - 0.68)	0.63 (0.61 - 0.64)	0.7 (0.68 - 0.71)
Zhang "poor"	Deterioration (in-hospital)	0.69 (0.67 - 0.71)	0.67 (0.65 - 0.68)	0.67 (0.66 - 0.69)	0.67 (0.66 - 0.69)
NEWS2	Deterioration (1 day)	0.77 (0.75 - 0.78)	0.66 (0.65 - 0.68)	0.75 (0.73 - 0.76)	0.62 (0.6 - 0.64)
Zhang "death"	Mortality (in-hospital)	0.72 (0.7 - 0.74)	0.66 (0.64 - 0.67)	0.7 (0.68 - 0.71)	0.64 (0.63 - 0.66)
Lu	Mortality (12 days)	0.56 (0.55 - 0.58)	0.64 (0.63 - 0.66)	0.59 (0.58 - 0.6)	0.66 (0.65 - 0.68)
qSOFA	Mortality (in-hospital)	0.67 (0.66 - 0.69)	0.61 (0.6 - 0.63)	0.66 (0.64 - 0.67)	0.59 (0.58 - 0.61)
MEWS	Deterioration (in-hospital)	0.72 (0.7 - 0.73)	0.6 (0.58 - 0.61)	0.68 (0.67 - 0.7)	0.56 (0.54 - 0.57)

Supplementary Table 6: Validation in London data excluding patients still hospitalised at the end of follow-up as sensitivity analysis (n = 7,913 participants).

Models are shown for prediction of in-hospital clinical deterioration and are sorted by C-statistic. CITL = calibration-in-the-large. 'Original outcome' column indicates original intended outcome for each candidate model during development. CITL and slopes are not shown for points score models since they are not on probability scale.

**Score**	**Original outcome**	**C-statistic**	**CITL**	**Slope**
4C Deterioration	Deterioration (in-hospital)	0.77 (0.76 - 0.78)	0.09 (0.04 - 0.14)	0.98 (0.92 - 1.03)
4C Mortality	Mortality (in-hospital)	0.69 (0.68 - 0.7)		
NEWS2	Deterioration (1 day)	0.69 (0.68 - 0.7)		
Zhang "poor"	Deterioration (in-hospital)	0.68 (0.67 - 0.69)	0.66 (0.6 - 0.72)	0.14 (0.11 - 0.17)
Zhang "death"	Mortality (in-hospital)	0.68 (0.66 - 0.69)	2.36 (2.3 - 2.42)	0.14 (0.1 - 0.18)
REMS	Mortality (in-hospital)	0.67 (0.66 - 0.68)		
DS-CURB65	Mortality (30 days)	0.67 (0.66 - 0.68)		
A-DROP	Mortality (30 days)	0.66 (0.65 - 0.67)		
CURB65	Mortality (30 days)	0.66 (0.65 - 0.67)		
qSOFA	Mortality (in-hospital)	0.63 (0.62 - 0.64)		
MEWS	Deterioration (in-hospital)	0.63 (0.61 - 0.64)		
Lu	Mortality (12 days)	0.62 (0.61 - 0.63)		

Supplementary Table 7: Validation in London data stratified by (a) community (n = 7,771 participants) vs (b) nosocomial infection (n = 468 participants) as sensitivity analysis.

Models are shown for prediction of in-hospital clinical deterioration and are sorted by C-statistic. CITL = calibration-in-the-large. 'Original outcome' column indicates original intended outcome for each candidate model during development. CITL and slopes are not shown for points score models since they are not on probability scale.

(a)

Score	Original outcome	C-statistic	CITL	Slope
4C Deterioration	Deterioration (in-hospital)	0.77 (0.76 - 0.78)	-0.02 (-0.07 - 0.04)	0.96 (0.91 - 1.02)
NEWS2	Deterioration (1 day)	0.7 (0.68 - 0.71)		
4C Mortality	Mortality (in-hospital)	0.68 (0.67 - 0.69)		
Zhang "death"	Mortality (in-hospital)	0.68 (0.66 - 0.69)	2.21 (2.14 - 2.27)	0.15 (0.11 - 0.19)
Zhang "poor"	Deterioration (in-hospital)	0.67 (0.66 - 0.69)	0.5 (0.44 - 0.57)	0.14 (0.11 - 0.17)
REMS	Mortality (in-hospital)	0.67 (0.66 - 0.68)		
DS-CURB65	Mortality (30 days)	0.66 (0.65 - 0.67)		
A-DROP	Mortality (30 days)	0.65 (0.64 - 0.66)		
CURB65	Mortality (30 days)	0.65 (0.64 - 0.66)		
MEWS	Deterioration (in-hospital)	0.63 (0.62 - 0.65)		
qSOFA	Mortality (in-hospital)	0.63 (0.62 - 0.64)		
Lu	Mortality (12 days)	0.62 (0.6 - 0.63)		

(b)

Score	Original outcome	C-statistic	CITL	Slope
4C Deterioration	Deterioration (in-hospital)	0.73 (0.68 - 0.78)	0.32 (0.12 - 0.53)	0.97 (0.68 - 1.26)
4C Mortality	Mortality (in-hospital)	0.69 (0.63 - 0.75)		
Zhang "poor"	Deterioration (in-hospital)	0.66 (0.61 - 0.71)	0.9 (0.63 - 1.17)	0.05 (-0.03 - 0.13)
DS-CURB65	Mortality (30 days)	0.66 (0.6 - 0.71)		
Zhang "death"	Mortality (in-hospital)	0.66 (0.6 - 0.71)	2.85 (2.57 - 3.12)	0.04 (-0.04 - 0.12)
Lu	Mortality (12 days)	0.65 (0.6 - 0.7)		
A-DROP	Mortality (30 days)	0.65 (0.6 - 0.7)		
NEWS2	Deterioration (1 day)	0.65 (0.6 - 0.7)		
CURB65	Mortality (30 days)	0.63 (0.58 - 0.69)		
REMS	Mortality (in-hospital)	0.61 (0.56 - 0.66)		
MEWS	Deterioration (in-hospital)	0.6 (0.55 - 0.66)		
qSOFA	Mortality (in-hospital)	0.59 (0.54 - 0.63)		

Supplementary Table 8: Validation parameters of prognostic model among community-acquired cases in London, excluding those with symptom onset recorded after admission date, as sensitivity analysis (n = 7,167 participants).

Models are shown for prediction of in-hospital clinical deterioration and are sorted by C-statistic. CITL = calibration-in-the-large. 'Original outcome' column indicates original intended outcome for each candidate model during development. CITL and slopes are not shown for points score models since they are not on probability scale.

**Score**	**Original outcome**	**C-statistic**	**CITL**	**Slope**
4C Deterioration	Deterioration (in-hospital)	0.77 (0.75 - 0.78)	-0.02 (-0.07 - 0.04)	0.96 (0.9 - 1.01)
NEWS2	Deterioration (1 day)	0.69 (0.68 - 0.7)		
4C Mortality	Mortality (in-hospital)	0.67 (0.66 - 0.69)		
Zhang "death"	Mortality (in-hospital)	0.67 (0.66 - 0.69)	2.23 (2.17 - 2.3)	0.14 (0.1 - 0.18)
Zhang "poor"	Deterioration (in-hospital)	0.67 (0.66 - 0.68)	0.55 (0.49 - 0.61)	0.13 (0.1 - 0.17)
REMS	Mortality (in-hospital)	0.67 (0.65 - 0.68)		
DS-CURB65	Mortality (30 days)	0.66 (0.65 - 0.67)		
CURB65	Mortality (30 days)	0.65 (0.64 - 0.66)		
A-DROP	Mortality (30 days)	0.65 (0.63 - 0.66)		
qSOFA	Mortality (in-hospital)	0.63 (0.62 - 0.64)		
MEWS	Deterioration (in-hospital)	0.63 (0.61 - 0.64)		
Lu	Mortality (12 days)	0.61 (0.6 - 0.62)		

Supplementary Table 9: Validation parameters of prognostic model in London cohort using alternative multiple imputation approach as sensitivity analysis (n = 8,239 participants).

Models are shown for prediction of in-hospital clinical deterioration and are sorted by C-statistic. CITL = calibration-in-the-large. 'Original outcome' column indicates original intended outcome for each candidate model during development. CITL and slopes are not shown for points score models since they are not on probability scale.

**Score**	**Original outcome**	**C-statistic**	**CITL**	**Slope**
4C Deterioration	Deterioration (in-hospital)	0.76 (0.75 - 0.77)	-0.01 (-0.06 - 0.04)	0.94 (0.88 - 1)
NEWS2	Deterioration (1 day)	0.69 (0.67 - 0.7)		
4C Mortality	Mortality (in-hospital)	0.67 (0.66 - 0.69)		
REMS	Mortality (in-hospital)	0.66 (0.65 - 0.67)		
DS-CURB65	Mortality (30 days)	0.65 (0.64 - 0.67)		
Zhang "death"	Mortality (in-hospital)	0.65 (0.64 - 0.67)	2.05 (1.96 - 2.13)	0.1 (0.08 - 0.13)
Zhang "poor"	Deterioration (in-hospital)	0.65 (0.63 - 0.66)	0.31 (0.24 - 0.39)	0.1 (0.08 - 0.12)
CURB65	Mortality (30 days)	0.64 (0.63 - 0.66)		
A-DROP	Mortality (30 days)	0.64 (0.63 - 0.65)		
qSOFA	Mortality (in-hospital)	0.63 (0.61 - 0.64)		
MEWS	Deterioration (in-hospital)	0.62 (0.61 - 0.63)		
Lu	Mortality (12 days)	0.61 (0.6 - 0.62)		

Supplementary Table 10: Validation parameters of single continuous predictors included in 4C Deterioration prognostic model in London cohort (n = 8,239 participants).

Shown for prediction of in-hospital clinical deterioration and sorted by C-statistic.

**Predictor**	**C-statistic**
C-reactive protein (mg/L)	0.68 (0.66 - 0.69)
Respiratory rate (per min)	0.62 (0.61 - 0.64)
Urea (mmol/L)	0.62 (0.61 - 0.64)
SpO2 on admission (%)	0.62 (0.61 - 0.63)
Age (years)	0.58 (0.56 - 0.59)
Lymphocytes (x10^9/L)	0.57 (0.56 - 0.59)

References

1 Harrell Jr FE. rms: Regression Modeling Strategies. 2019.

2 White IR, Royston P, Wood AM. Multiple imputation using chained equations: Issues and guidance for practice. *Stat Med* 2011; **30**: 377–99.

3 Buuren S van, Groothuis-Oudshoorn K. mice: Multivariate Imputation by Chained Equations in R. *J Stat Softw* 2011; **45(3)**: 1–67.

4 Rubin DB. Multiple imputation for nonresponse in surveys. Wiley-Interscience, 2004.

5 Robin X, Turck N, Hainard A, *et al.* pROC: an open-source package for R and S+ to analyze and compare ROC curves. *BMC Bioinformatics* 2011; **12**: 77.

6 Wickham H, Averick M, Bryan J, *et al.* Welcome to the Tidyverse. *J Open Source Softw* 2019; **4**: 1686.

7 Brown M. rmda: Risk Model Decision Analysis. 2018.

8 Singh K. runway: Evaluation and Comparison of Prediction Models. 2020.

9 Seymour CW, Liu VX, Iwashyna TJ, *et al.* Assessment of Clinical Criteria for Sepsis. *JAMA* 2016; **315**: 762.

10 Royal College of Physicians. National Early Warning Score (NEWS) 2 | RCP London. https://www.rcplondon.ac.uk/projects/outputs/national-early-warning-score-news-2 (accessed July 1, 2020).

11 Lim WS, Eerden MM van der, Laing R, *et al.* Defining community acquired pneumonia severity on presentation to hospital: an international derivation and validation study. *Thorax* 2003; **58**: 377–82.

12 Riley RD, Snell KI, Ensor J, *et al.* Minimum sample size for developing a multivariable prediction model: PART II - binary and time-to-event outcomes. *Stat Med* 2019; **38**: 1276–96.

13 Riley RD, Ensor J, Snell KIE, *et al.* Calculating the sample size required for developing a clinical prediction model. *BMJ* 2020; **368**: m441.
